# Perivascular adipose tissue from female rats fed a high‐fat diet impaired mesenteric artery vasodilation

**DOI:** 10.14814/phy2.70746

**Published:** 2026-01-28

**Authors:** Gurneet S. Sangha, Ryan M. Sapp, Callie M. Weber, Donaysia Torbit, Nimisha Rangachar, Anne Barnes, Alisa M. Clyne

**Affiliations:** ^1^ Fischell Department of Bioengineering University of Maryland College Park College Park Maryland USA; ^2^ Department of Biomedical Engineering University of California Irvine California USA; ^3^ School of Medicine, Department of Orthopedic Surgery Stanford University Stanford California USA

**Keywords:** cardiovascular, endothelial dysfunction, high‐fat diet, inflammation, obesity, Perivascular adipose tissue, sex differences, vasodilation

## Abstract

High‐fat diet (HFD) is a cardiovascular risk factor that may disproportionately affect women compared to men. HFD alters perivascular adipose tissue (PVAT), which surrounds arteries and regulates vascular function. This study investigated the sex‐specific effects of HFD on PVAT‐mediated vascular dysfunction. Male and female Sprague‐Dawley rats were fed a normal chow diet (NCD) or HFD for 16 weeks. Thoracic aortic PVAT was isolated, and PVAT adipokine expression and inflammatory markers were measured. We then assessed PVAT‐conditioned media effects on vasodilation, endothelial oxidative stress, and endothelial nitric oxide (NO) synthase. PVAT‐conditioned media from HFD female but not male rats impaired acetylcholine‐induced vasodilation in mesenteric arteries; however, it did not increase oxidative stress or decrease endothelial NO synthase activation in rat endothelial cells in vitro. PVAT from HFD female rats had higher CD68 expression and acetyl‐NF‐κB/NF‐κB ratio than PVAT from NCD female rats. Overall, HFD caused a greater PVAT‐induced impairment in vasodilation in female as compared to male rats, which aligns with the heightened cardiovascular risk in women consuming a HFD.

## 1 INTRODUCTION

Cardiovascular disease (CVD) is the leading cause of death in both sexes (Martin et al., 2024). In spite of this fact, CVD research has historically centered on male physiology, overlooking sex differences in CVD mechanisms (Merz & Cheng, 2016; Regitz‐Zagrosek & Kararigas, 2016). For example, studies using primarily male subjects have shown that a high‐fat diet (HFD), such as the prevalent “Western diet,” increases CVD risk (Duan et al., 2018; Gioscia‐Ryan et al., 2021; Hu et al., 2000; Kopp, 2019; Rakhra et al., 2020). However, several studies that analyzed men and women separately found a higher risk of CVD in women who consumed a HFD (Jakobsen et al., 2004; Wilson et al., 2002). In a 16‐year follow‐up study of more than 3500 Danish men and women, a 5% increase in energy intake due to saturated fat was associated with a 36% increase in coronary heart disease risk in women, while men had no increase in risk (Jakobsen et al., 2004). Thus, there remains a clinical need to investigate the unique pathways driving CVD in men and women consuming a HFD to inform the development of sex‐specific preventative strategies.

The endothelium governs vascular health largely through the production of nitric oxide (NO), a key vasodilator (Green et al., 2014; Heiss et al., 2015; Matsuzawa et al., 2017; Vanhoutte et al., 2017). Reduced NO bioavailability, either by diminished production or excess scavenging, is a hallmark of endothelial dysfunction and contributes to CVD (Vanhoutte et al., 2017). Mechanical stimuli like shear stress and biochemical signals such as acetylcholine elevate cytosolic Ca^2+^, activating endothelial nitric oxide synthase (eNOS) to enhance NO production (Davies et al., 2019; Wang et al., 2016). Conversely, elevated oxidative stress due to reactive oxygen species (ROS) production decreases NO bioavailability by converting NO into peroxynitrite (Pacher et al., 2007). The balance between NO production and NO scavenging is essential to vascular health.

Perivascular adipose tissue (PVAT) surrounds almost all arteries and has emerged as an important paracrine regulator of endothelial NO bioavailability (Cheng et al., 2018; Xia & Li, 2017). Healthy PVAT supports vasodilation, as demonstrated by less norepinephrine‐induced contraction in rat aorta with intact PVAT than in PVAT‐free aorta (Soltis & Cassis, 1991). PVAT produces NO using its own functional eNOS, and it releases adipokines (e.g., adiponectin) that stimulate endothelial NO production and vasodilation. These properties are referred to as an “anti‐contractile” PVAT effect.

HFD impairs anti‐contractile PVAT effects in rodents through inflammation and oxidative stress, eNOS dysfunction, and altered adipokine secretion. These pathological changes lead to reduced endothelium‐dependent vasodilation in response to acetylcholine (Aoqui et al., 2014; Gil‐Ortega et al., 2014; Horimatsu et al., 2018; Ma et al., 2010; Sousa et al., 2019; Victorio et al., 2021; Wang et al., 2012; Xia et al., 2016; Xia et al., 2017) and increased smooth muscle cell contraction to phenylephrine or norepinephrine (Aghamohammadzadeh et al., 2016; Aoqui et al., 2014; da Costa et al., 2017; Han et al., 2018; Ma et al., 2010; Victorio et al., 2021). HFD stimulates PVAT to secrete inflammatory cytokines that increase ROS production and NO scavenging (Almabrouk et al., 2018; Aoqui et al., 2014; Han et al., 2018; Sousa et al., 2019; Xia et al., 2017). For example, HFD mice deficient in P‐selectin glycoprotein ligand‐1, a mediator of macrophage recruitment, showed reduced macrophage PVAT infiltration and preserved mesenteric artery vasodilation to acetylcholine (Wang et al., 2012). HFD also impairs vascular function by shifting eNOS activity towards ROS generation instead of NO production. This effect was demonstrated in HFD C57Bl/6J mice, in which PVAT treatment with the eNOS inhibitor NG‐nitro‐L‐arginine methyl ester (L‐NAME) reduced ROS production (Xia et al., 2016). Lastly, HFD impairs PVAT secretion of adipokines that support NO production, particularly adiponectin (Aghamohammadzadeh et al., 2016; Almabrouk et al., 2018; Aoqui et al., 2014; Han et al., 2018). RNA interference of APPL1, which interacts with adiponectin receptor AdipoR1 and ‐R2, attenuated adiponectin‐induced phosphorylation at eNOS (Ser1177), a marker of eNOS activation (Cheng et al., 2007). These interrelated mechanisms reduced vasodilation and increased vasoconstriction, highlighting the complex pathophysiology of HFD‐induced PVAT dysfunction.

Most PVAT studies primarily focus on male animals (Aghamohammadzadeh et al., 2016; Almabrouk et al., 2018; Aoqui et al., 2014; da Costa et al., 2017; Gil‐Ortega et al., 2014; Han et al., 2018; Horimatsu et al., 2018; Ma et al., 2010; Sousa et al., 2019; Victorio et al., 2021; Wang et al., 2012; Xia et al., 2016; Xia et al., 2017), even though animal models show sex‐ and hormone‐dependent differences in adipose tissue development, metabolism, inflammation, and adipokine secretion (Kuryłowicz, 2023; Steiner & Berry, 2022). Emerging evidence shows that PVAT from HFD female rodents may exhibit more pronounced inflammation and deleterious effects on vascular function. For instance, mesenteric and thoracic PVAT from female Dahl S rats fed a HFD for 10 weeks contained more M2‐like macrophages and fewer memory T cells than PVAT from male rats fed a HFD, with these sex differences becoming more pronounced after 24 weeks (Kumar et al., 2021). Additionally, PVAT anti‐contractile effects decreased earlier in female C57Bl6/J mice fed a HFD than in age‐matched males (Victorio et al., 2021). These studies indicate that female animals may be more susceptible to HFD‐induced PVAT changes and associated vascular complications.

**FIGURE 1:**
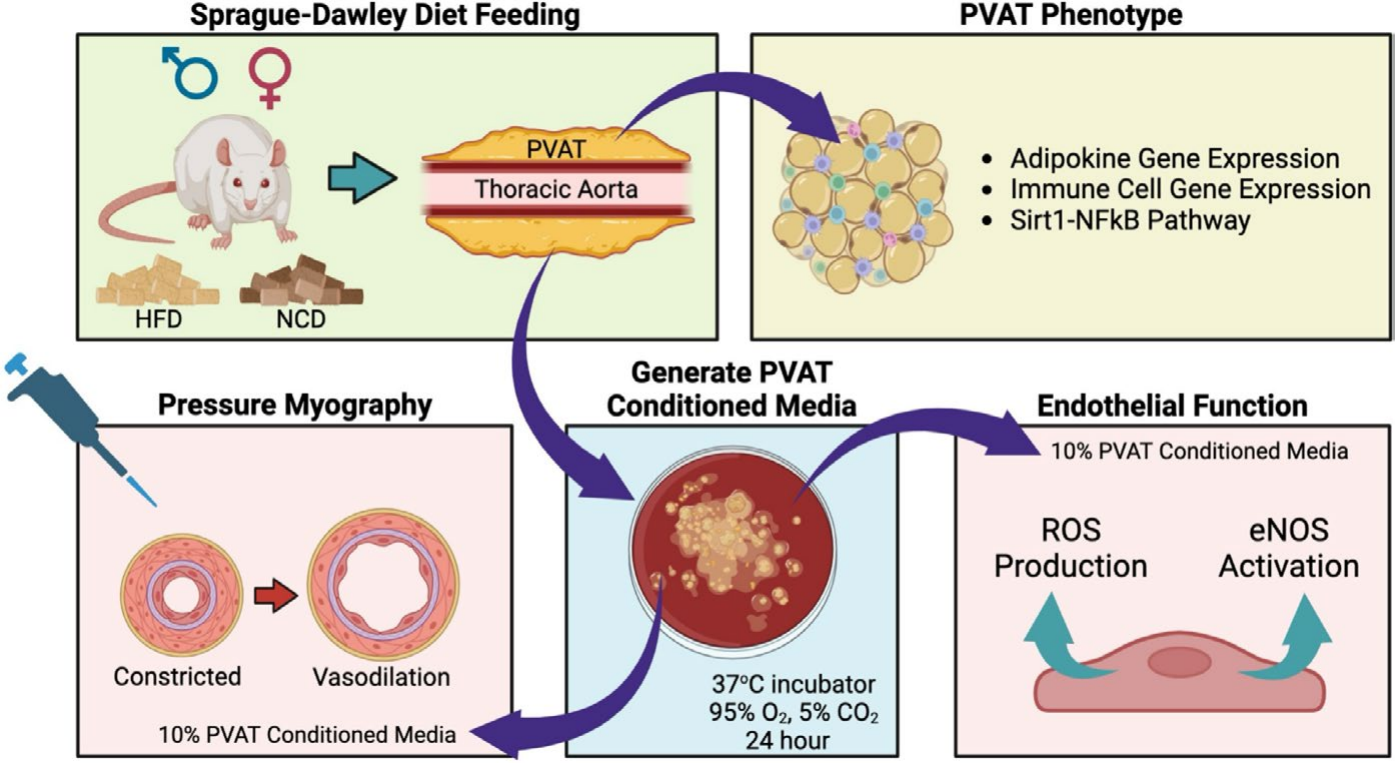
Summary of experimental methods. Thoracic PVAT from rats fed a HFD or NCD for 16 weeks was excised and used for PVAT phenotyping or incubated in media for 24 h. Pressure myography was used to quantify the effects of PVAT‐conditioned media on mesenteric artery vasodilation. Finally, we determined how PVAT‐conditioned media affects endothelial ROS production and eNOS activation as markers for NO bioavailability.

The mechanisms by which HFD differentially affects male and female PVAT anti‐contractile effects remain unclear. We hypothesized that PVAT from HFD rats would reduce vasodilation more in female rats than in male rats. We fed male and female rats either a normal chow diet (NCD) or HFD for 16 weeks and examined PVAT phenotype (Figure 1). We then investigated how thoracic PVAT from HFD male and female rats influenced vasodilation using pressure myography, which closely mimics in vivo vasodilation and the crosstalk between PVAT and arteries. We used mesenteric arteries to measure vasodilation because we cannot perform pressure myography on the aorta due to branching vessels. Finally, we investigated how PVAT affects NO production and ROS in endothelial cells in vitro. This work highlights the importance of PVAT sex differences in HFD‐induced vascular dysfunction and may lead to new insights into why HFD is associated with a higher risk of CVD in women.

## 2 METHODS

### 2.1 Animals

All animal procedures were approved by the University of Maryland Institutional Animal Care and Use Committee (Project ID# 1643983‐8). Male and female Sprague–Dawley rats were acquired from Hilltop Lab Animals. Rats were used because their PVAT quantity enabled us to generate enough conditioned media for our experiments. Rats were housed in the University of Maryland vivarium in ventilated cages on soft pelleted bedding with a 12 h light/12 h dark cycle. Room temperature was 69–75°F. Rats were provided with drinking water via Hydropac pouches, and two rats of the same sex were in each cage. At 6 weeks of age, rats were either maintained on a normal chow diet (NCD; 14%–18% kcal from fat, 58%–65% kcal from carbohydrates, 21%–24% kcal from protein) or were switched to a high‐fat diet (HFD; 45% kcal from fat, 35% kcal from carbohydrates, 20% kcal from protein; Research Diets, Diet #D12451) ad libitum for 16 weeks. The increased fat content in the HFD was from lard.

### 2.2 Euthanasia and tissue collection

All rats were weighed weekly and euthanized at 22 weeks of age. Euthanasia was performed by anesthetizing animals using 5% isoflurane and then exsanguinating animals via cardiac puncture. The mesentery was removed and placed in ice‐cold physiological saline solution (PSS) containing 0.1% bovine serum albumin (BSA; MilliporeSigma; A7906). Fresh PSS composed of 119.1 mM NaCl, 4.7 mM KCL, 2.4 mM MgSO_4_, 1.2 mM KH_2_PO_4_, 10 mM HEPES, 2.8 mM CaCl_2_, 14.9 mM NaHCO_3_, 5.5 mM glucose, and 68.4 mM EDTA was prepared daily. PSS was pH balanced to 7.4 before use. After carefully removing the surrounding fat, third‐order mesenteric arteries were isolated and placed in fresh, ice‐cold PSS containing 0.1% BSA for use in pressure myography experiments.

All visible PVAT surrounding the thoracic aorta, extending along the spine to the diaphragm, was completely excised and placed in phosphate‐buffered saline (PBS; ThermoFisher Scientific; 70011069) on ice. The PVAT was then weighed and cut into ~10 mg pieces. For later RNA analysis, 30 mg PVAT was placed in RNAlater (Millipore Sigma; R0901), stored at 4°C for 24 h, and then at −20°C until needed. For later protein analysis, 25 mg PVAT was flash‐frozen in liquid nitrogen and stored at −80°C.

The remaining PVAT was placed in Dulbecco's Modified Eagle's Medium (DMEM; Corning; 10‐014‐CV) supplemented with 1% penicillin–streptomycin (ThermoFisher Scientific; 15140163) at 160 mg PVAT per 1 mL media and cultured for 24 h at 37°C, 5% CO_2_ to generate PVAT‐conditioned media. After 24 h, the PVAT‐conditioned media was collected, centrifuged at 2500 × g for 20 min to remove debris, aliquoted, and stored at −80°C until use.

### 2.3 Pressure Myography

We tested the effects of PVAT‐secreted paracrine factors on arterial vasodilation using pressure myography. While wire myography is often used in PVAT studies, wire myography exposes both the endothelium and smooth muscle cells to PVAT‐conditioned media and does not allow the artery to be pressurized. We used pressure myography to expose only the outside of the blood vessel to PVAT‐conditioned media and thereby better mimic PVAT‐arterial crosstalk, as well as to better mimic in vivo hemodynamics by maintaining arterial pressure. All pressure myography experiments were performed on third‐order mesenteric arteries, since the thoracic aorta contains numerous branch vessels which make it unsuitable for pressure myography. On the day that rats were euthanized, mesenteric arteries were pressurized and incubated for 30 min with or without PVAT‐conditioned media from rats of the same sex and diet. For example, mesenteric arteries from a NCD female rat were treated with PSS ± 10% PVAT‐conditioned media from a previously euthanized NCD female rat.

Mesenteric arteries were cannulated on 300 μm steel cannulas and superfused with PSS in a myograph chamber (DMT; 114P). The PSS in the myograph chamber was maintained at 37°C and continuously bubbled with carbogen (5% CO_2_, 95% O_2_). An inverted microscope (Zeiss; Axio Vert.A1) was used to visualize and track artery diameter using wall‐tracking software (Myoview 4.0). Arterial pressure was progressively increased from 0 to 60 mmHg in 10 mmHg increments over 30 min. We then tested mesenteric artery viability by constricting the vessel using 10^−5^ M phenylephrine (Acros Organics; 207240100). Arteries that constricted >10% were considered viable for our study. Following viability testing, the myograph chamber was washed and the PSS was replaced with either untreated PSS or 10% PVAT‐conditioned media diluted in PSS for 30 min.

Finally, we measured mesenteric artery vasoreactivity by quantifying endothelial‐dependent and independent vasodilation. After establishing a baseline arterial diameter, the vessel was constricted again using 10^−5^ M phenylephrine. The artery was stabilized for 5 min before endothelial‐dependent vasodilation was measured by adding increasing acetylcholine (10^−9^ M–10^−5^ M; MilliporeSigma; A6625) to the myograph chamber. The chamber was then washed, and endothelial‐independent vasodilation was measured using increasing NONOate (10^−9^ M–10^−4^ M; Cayman Chemical Company; 82100). Vasodilation was measured using the maximal steady‐state baseline artery diameter (D_m_), steady‐state diameter after phenylephrine preconstriction (D_p_), and maximum steady‐state diameter after acetylcholine or NONOate treatment (D_a_) as:

$$\text{Vasodilation}\;\left(\%\right)=\left[\frac{D_{a}-D_{p}\;}{D_{m}-D_{p}}\right]x\;100$$

Data between groups were statistically compared using pEC50. EC50 is the acetylcholine dose needed to produce half‐maximal vasodilation, and pEC50 is the negative logarithm of the EC50 value.

### 2.4 RNA analysis

PVAT samples stored in RNAlater were rinsed in ice‐cold PBS and moved to 700 μL ice‐cold Qiazol solution (Qiagen; 79306). Samples were homogenized on ice using a rotor‐stator tissue homogenizer (Benchmark Scientific D1000). RNA was isolated from homogenized PVAT samples using the miRNeasy mini kit (Qiagen; 217004) according to the manufacturer's instructions. RNA was isolated from RAEC lysates using the trizol – chloroform method (Curtis et al., 2025). A spectrophotometer (ThermoFisher Scientific; Nanodrop 2000c) was used to determine RNA concentration, and an equal amount of RNA from each sample was reverse transcribed using a High‐Capacity cDNA Reverse Transcription Kit (ThermoFisher Scientific; 4368813).

Gene expression was determined via real‐time quantitative polymerase chain reaction (RT‐qPCR) on a Quantstudio 7 Flex Real‐Time PCR system (ThermoFisher Scientific) using TaqMan™ Fast Advanced Master Mix (ThermoFisher Scientific; 4444963). Gene‐specific Taqman primers (ThermoFisher Scientific) were used to quantify the expression of leptin (Rn00565158_m1), adiponectin (Rn00595250_m1), and CD68 (Rn01495634_g1). Samples were analyzed for each gene in duplicate. Gene expression levels were determined using the 2^−∆∆CT^ method (where CT is the cycle threshold) of relative quantification.

### 2.5 Enzyme‐linked immunosorbent assay

Leptin concentrations were measured using an Enzyme‐Linked Immunosorbent Assay (ELISA; RayBiotech; Catalogue #ELR‐Leptin‐1). Plasma samples were diluted three‐fold with the appropriate assay diluent. Samples were assayed in duplicate. Immediately after adding stop solution, absorbance was measured at 450 nm via a spectrophotometer (Tecan; Spark Multimode Microplate Reader).

### 2.6 Cell culture

Rat aortic endothelial cells (RAEC) pooled from male and female rats (Angio‐Proteomie, cAP‐r0001) were maintained at 37°C, 5% CO_2_, and used for experiments at passages 6–8. Cells were grown in endothelial growth medium‐2 (EGM‐2; Lonza; CC‐3162) supplemented with 10% fetal bovine serum (FBS; HyClone; SH30088), 1% penicillin–streptomycin (ThermoFisher Scientific; 15140163), and 1% L‐glutamine (ThermoFisher Scientific; 25030081). RAEC were seeded into 6‐well plates for eNOS phosphorylation experiments and 24‐well plates for oxidative stress experiments. Upon reaching 80%–90% confluency, cells were washed with PBS and treated with EGM‐2 supplemented with 5% FBS and 10% PVAT‐conditioned media for 30 min. For eNOS phosphorylation experiments, RAEC were also treated with 2 μM Yoda1, a piezo1 agonist, for an additional 30 min to activate eNOS.

### 2.7 Oxidative stress assay

Confluent RAEC were first treated with 0 or 10% PVAT conditioned media or tert‐butyl hydroperoxide (TBHP; positive control; ThermoFisher Scientific; I36007) in DMEM for 30 min at 37°C. Cells were washed once with Hank's Balanced Salt Solution with calcium and magnesium (HBSS; Millipore Sigma; 55037C) and then incubated with 25 μM carboxy‐H_2_DCFDA (ThermoFisher Scientific; I36007) containing 1 μM Hoechst (ThermoFisher Scientific; 62249) for 30 min at 37°C. Carboxy‐H_2_DCFDA is taken up by cells and then oxidized by ROS to produce a fluorescent signal. Finally, RAEC were washed twice with HBSS before imaging at 10× magnification on a confocal laser scanning microscope (Nikon Eclipse Ti2‐E). FIJI (NIH) (Nishiyama et al., 2017) was used to quantify fluorescence by subtracting background intensity from all ROS and nuclei images using a rolling ball algorithm, followed by calculating the average fluorescence intensity in ROS images and cell count in nuclei images. The average ROS intensity was then normalized to cell count.

### 2.8 Western blots

PVAT samples stored at −80°C were transferred to ice‐cold Radioimmunoprecipitation Assay buffer (RIPA; ThermoFisher Scientific; 89901) supplemented with Halt protease and phosphatase inhibitor cocktail (ThermoFisher Scientific; 78440). Samples were homogenized on ice using a rotor‐stator tissue homogenizer and centrifuged to collect protein. RAEC were washed twice with ice‐cold PBS, lysed in RIPA supplemented with Halt protease and phosphatase inhibitor cocktail, and centrifuged for 15 min at 17,000 × g, 4°C to remove cell debris. PVAT and RAEC protein samples were normalized by protein concentration using a Bicinchoninic acid (BCA) assay (ThermoFisher Scientific; 23225). Samples were separated by sodium dodecyl‐sulfate polyacrylamide gel electrophoresis (SDS‐PAGE) in 4%–12% bis‐tris gels (ThermoFisher Scientific; NP0323BOX) and transferred to PVDF membranes (ThermoFisher Scientific; IB24001) using an iBlot 2 (ThermoFisher Scientific; IB21001). Membranes were blocked for 1 h with tris‐buffered saline (TBS; Fisher Scientific; BP24711) containing 1% Tween‐20 (TBS‐T; ThermoFisher Scientific; 85115) and either 5% BSA or 5% nonfat powdered milk. After washing, membranes were incubated overnight at 4°C with primary antibodies for eNOS (Cell Signaling Technology; 9572), phosphorylated‐eNOS‐Ser1177 (Cell Signaling Technology; 9571), phosphorylated‐eNOS‐Thr495 (Cell Signaling Technology; 9574), SIRT1 (Cell Signaling Technology; 9475), NF‐κB (Cell Signaling Technology; 8242), acetylated‐NF‐κB (Lys310; ThermoFisher Scientific; PA5‐17264), or GAPDH (Cell Signaling Technology; 2118) diluted 1:1000 in TBS‐T with either 1% BSA or 5% milk. Membranes were exposed to the appropriate horseradish peroxidase‐conjugated secondary antibody (Promega) diluted 1:2000 for 2 h at room temperature, after which membranes were imaged on an Alpha Innotech Fluorchem Imager using a chemiluminescence kit (SuperSignal West Pico PLUS, ThermoFisher Scientific; 34580). Protein band intensity was quantified using ImageJ (NIH) or AlphaView and normalized to GAPDH.

### 2.9 Statistical analyses

All experiments had a sample size of at least five in each experimental group to comply with the guidelines set by Curtis et al. (Curtis et al., 2025), with the exception of pressure myography on male rat mesenteric arteries that were untreated or treated with HFD‐conditioned media from male rats (*n* = 4). In addition, sample sizes in the pressure myography experiments were not always equal; for instance, mesenteric arteries from female rats that were untreated (*n* = 9) or treated with NCD‐conditioned media (*n* = 7). In each case, samples were excluded due to the previously described pre‐established criteria, specifically < 10% constriction response to phenylephrine, indicating that the artery was not viable. Additional samples could not be added to the study due to limited conditioned media supply. Data collection for animal experiments was supervised by an experienced investigator who did not know the study hypothesis. This investigator ensured that data were collected consistently and correctly. PVAT analysis was conducted prior to pressure myography to avoid potential bias. All other experiments were conducted by researchers blinded to the pressure myography results, with the exception of the oxidative stress experiments, which did not show differences among experimental groups.

Data were analyzed with GraphPad Prism 10. When only two conditions were compared, a Mann–Whitney non‐parametric test was used to determine statistical significance. When the same animal or vessel was measured multiple times, a repeated measures ANOVA was used. When more than two conditions were compared for independent samples, a two‐way ANOVA was used. If a significant main effect or interaction was observed, a post‐hoc test was used to make comparisons among groups. Different post‐hoc tests were used depending on the specific comparisons. When samples were compared across sex or diet, a Fisher's LSD test was used. When samples were compared across sex, diet, and another treatment, a Tukey multiple comparisons test was used. The statistical analysis method for each data set is listed in the figure caption. *p* < 0.05 was considered significant. Data are represented as mean ± standard deviation.

## 3 RESULTS

### 3.1 Body and PVAT weight were not greater in Sprague Dawley rats fed a HFD versus NCD

**FIGURE 2:**
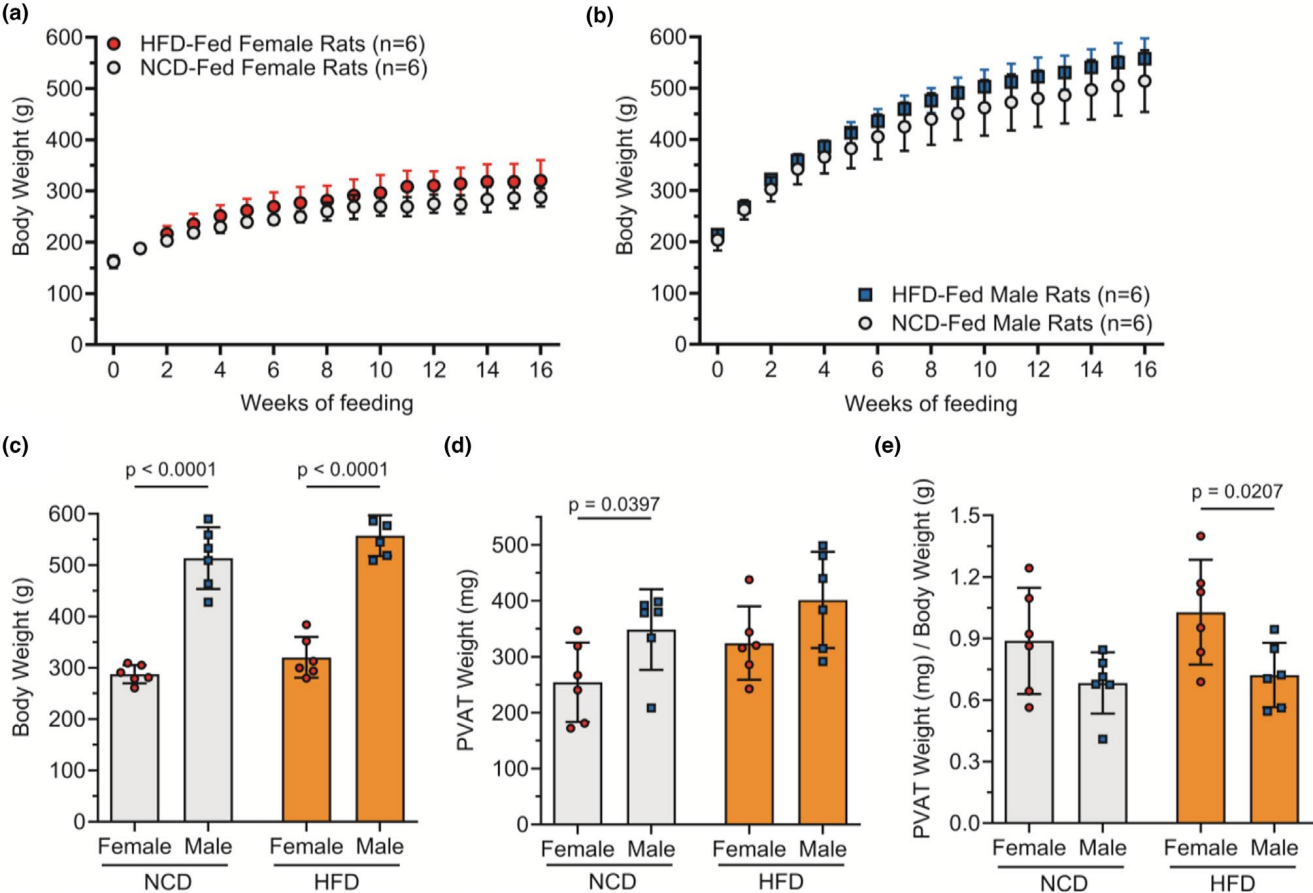
HFD did not significantly increase male and female Sprague Dawley rat body or PVAT weight over NCD. Body weight over 16 weeks of NCD or HFD feeding in female (a) and male (b) rats. Body weight at study endpoint in NCD or HFD female and male rats (c). Thoracic PVAT weight at study endpoint in NCD or HFD female and male rats (d). Thoracic PVAT weight per body weight at study endpoint in NCD or HFD female and male rats (e). *n* = 6 rats per group. Data represented as mean ± standard deviation. Statistical significance was determined using a repeated measures ANOVA (a, b) or a two‐way ANOVA followed by Fisher's LSD test (c–e).

We first determined how HFD affected rat body weight as a marker for obesity. Repeated measures ANOVA revealed a significant main effect of time (*p* < 0.0001) but no significant main effect of diet on rat body weight. Interaction between time and diet was significant in female rats (*p* = 0.037) but not in male rats (*p* = 0.085). Throughout the 16‐week feeding period, body weight significantly increased with time from 161.7 ± 12.1 g to 287.7 ± 17.9 g in NCD female rats and from 164 ± 8.5 g to 320.5 ± 39.6 g in HFD female rats (Figure 2a). Body weight also significantly increased with time in NCD male rats (203.8 ± 20.5 g to 513.8 ± 60.1 g) and in HFD male rats (214.3 ± 9.3 g to 557.5 ± 39.6 g); (Figure 2b). At the time of euthanasia (Figure 2c), two‐way ANOVA indicated a significant main effect of sex (*p* = 0.038) and diet (*p* < 0.0001) but no significant interaction between sex and diet (*p* = 0.76). Male rats weighed ~75% more than female rats (*p* < 0.0001). These results show that HFD did not significantly increase body weight in male or female rats compared to NCD.

Next, we tested how HFD affected PVAT weight (Figure 2d). Two‐way ANOVA indicated a significant main effect of sex (*p* = 0.010) and no significant main effect of diet (*p* = 0.56) or interaction between sex and diet (*p* = 0.78) on PVAT weight. NCD male rats had 37% higher thoracic PVAT weight than NCD female rats (*p* = 0.040). However, HFD male rats did not have greater thoracic PVAT weight than HFD female rats (*p* = 0.086). When we normalized PVAT weight to bodyweight (Figure 2e), two‐way ANOVA indicated a significant main effect of sex (*p* = 0.008), no significant main effect of diet (*p* = 0.32), and no significant interaction between sex and diet (*p* = 0.56). For normalized PVAT weight, HFD female rats had 43% higher normalized PVAT weight than male rats (*p* = 0.0207).

### 3.2 HFD increased some PVAT inflammatory markers in female but not male rats

**FIGURE 3:**
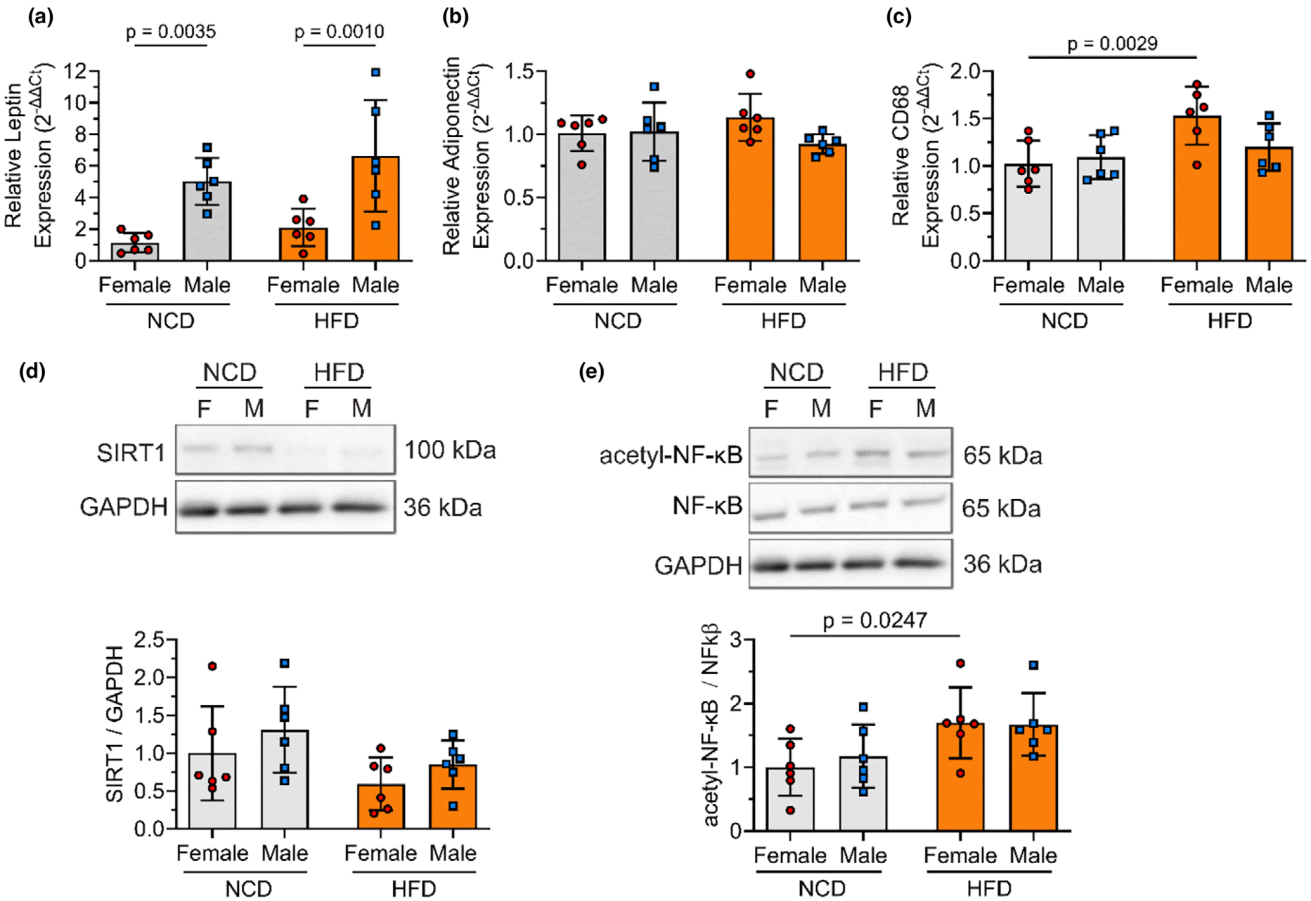
HFD increased some inflammatory biomarkers in PVAT from female rats compared to male rats. Relative gene expression of PVAT leptin (a), adiponectin (b), and CD68 (c). Representative Western blot and quantification of SIRT1 (d) and acetyl‐NF‐κB:NF‐κB ratio (e). *n* = 6 rats per group. Data are represented as mean ± standard deviation. Statistical significance was determined using a two‐way ANOVA followed by Fisher's LSD test. The full Western blots are provided in the Data S1.

We then investigated how HFD impacted PVAT adipokines and inflammatory markers. For PVAT leptin gene expression (Figure 3a), two‐way ANOVA indicated a significant main effect of sex (*p* < 0.0001) with no significant main effect of diet (*p* = 0.13) and no significant interaction between sex and diet (*p* = 0.70). PVAT leptin gene expression was more than 300% greater in male as compared to female NCD rats (*p* = 0.0035) and more than 200% higher in male rats as compared to female HFD rats (*p* = 0.0010). However, neither sex nor HFD increased leptin concentration in PVAT‐conditioned media (Figure S1; no significant effects by two‐way ANOVA). We also did not detect adiponectin gene expression differences in PVAT from male rats compared to female rats (Figure 3b; no significant effects by two‐way ANOVA). We measured circulating leptin concentration to determine how HFD affected systemic adipokine levels (Figure S2). Two‐way ANOVA indicated no significant main effect of sex (*p* = 0.12), a significant main effect of diet (*p* = 0.042), and no significant interaction between sex and diet (*p* = 0.19). HFD female rats had 175% higher plasma leptin (395.3 ± 227.7 pg/mL) than NCD female rats (142.7 ± 64.9 pg/mL; *p* = 0.0269). Our findings suggest that HFD elevated circulating leptin in female but not male rats.

CD68 gene expression was used to measure PVAT monocytes (Figure 3c). Two‐way ANOVA indicated no significant main effect of sex (*p* = 0.23), a significant main effect of diet (*p* = 0.0088), and no significance in the interaction between sex and diet (*p* = 0.073). Specifically, HFD significantly increased CD68 expression in female rats by 50% (*p* = 0.0029). SIRT1, an NAD^+^‐dependent protein deacetylase, reduces inflammation by deacetylating the p65 subunit of the NF‐κB complex. NF‐κB in its acetylated form activates pro‐inflammatory pathways (DeVallance et al., 2018; Hariri & Thibault, 2010). For anti‐inflammatory PVAT SIRT1 (Figure 3d), two‐way ANOVA indicated no significant main effect of sex (*p* = 0.1685), a significant main effect of diet (*p* = 0.0412), and no significance in the interaction between sex and diet (*p* = 0.898). For pro‐inflammatory acetyl‐NF‐κB:NF‐κB ratio (Figure 3e), two‐way ANOVA indicated no significant main effect of sex (*p* = 0.722), a significant main effect of diet (*p* = 0.0081), and no significance in the interaction between sex and diet (*p* = 0.6263). HFD increased the acetyl‐NF‐κB:NF‐κB ratio by 70% (*p* = 0.0247) in female rats. There were no statistically significant differences in total PVAT NF‐κB or acetyl‐NF‐κB (Figure S3), or in PVAT gene expression of the inflammatory cytokines IL‐6 or TNFα in female or male rats fed a NCD or HFD (Figure S4). These data suggest that HFD induced a larger pro‐inflammatory shift in PVAT from female as compared to male rats.

### 3.3 PVAT‐conditioned media from HFD female rats impaired endothelial‐dependent vasodilation

**FIGURE 4:**
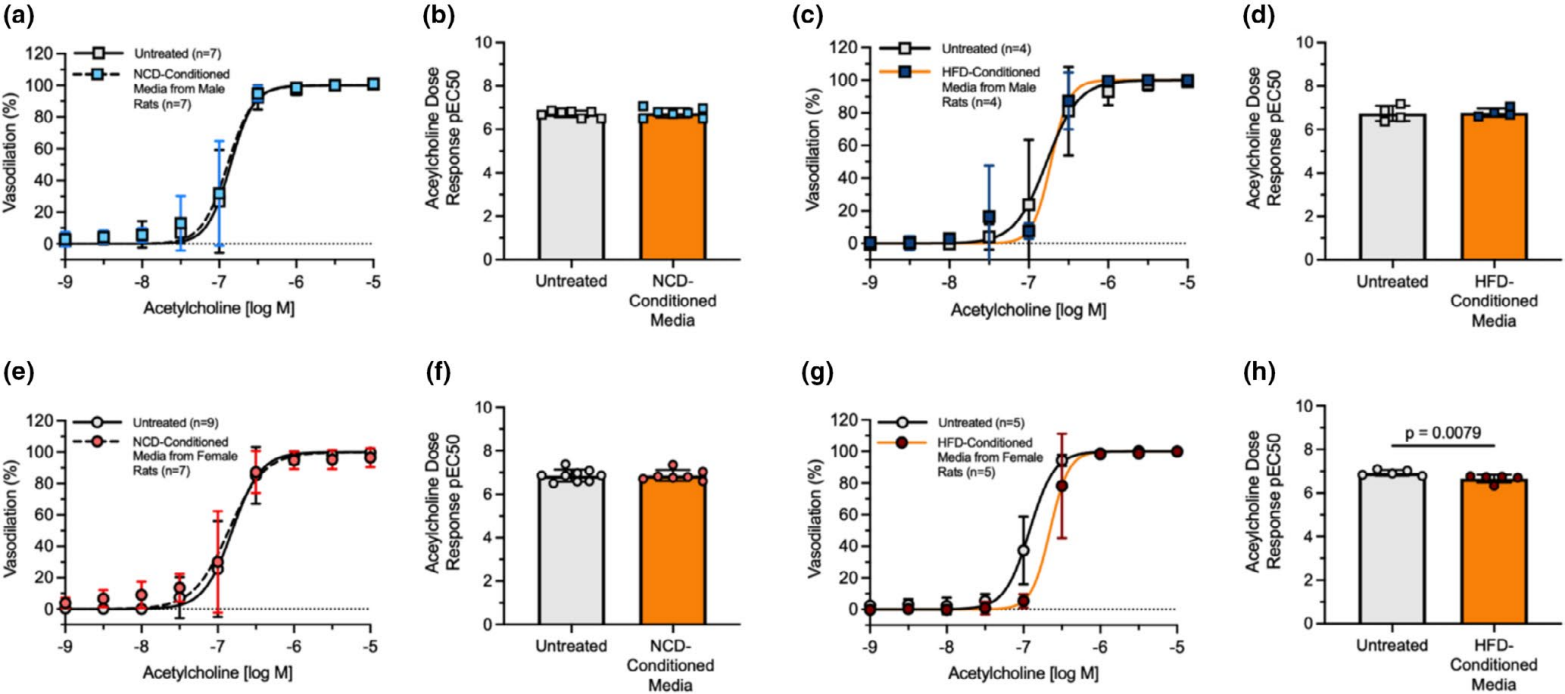
PVAT‐conditioned media from HFD female rats impaired mesenteric artery vasodilation. Pressure myography and pEC50 from mesenteric arteries undergoing acetylcholine dose–response after 30‐min treatment with PSS (untreated; *n* = 4–9) or 10% PVAT conditioned media from NCD male rats (*n* = 7; a, b), HFD male rats (*n* = 4; c, d), NCD female rats (*n* = 7; e, f), and HFD female rats (*n* = 5; g, h). PVAT conditioned media effects on vasodilation were tested on mesenteric arteries from sex and diet‐matched rats. Data are represented as mean ± standard deviation. Statistical significance for panels b, d, f, and h was determined using a Mann–Whitney test.

We subsequently used an acetylcholine‐dose response to study how NCD and HFD PVAT‐conditioned media affected male and female mesenteric artery vasodilation. Mesenteric arteries treated with PVAT‐conditioned media from HFD female rats experienced a rightward shift in acetylcholine dose response, decreasing the pEC50 from 6.93 in mesenteric arteries treated with PSS to 6.66 in mesenteric arteries treated with HFD PVAT‐conditioned media (*p* = 0.0079; Figure 4). We did not observe a significant difference in the acetylcholine dose–response or pEC50 in mesenteric arteries treated with PVAT‐conditioned media from NCD male rats, HFD male rats, or NCD female rats (Figure 4). We then checked to ensure that vasodilation did not change with other experimental parameters. Vasodilation was similar in mesenteric arteries from female (pEC50 = 6.88) and male rats (pEC50 = 6.72; *p* = 0.077; Figure S5). HFD did not impair acetylcholine‐induced vasodilation in male and female mesenteric arteries not treated with PVAT‐conditioned media (Figure S6). These results suggest that PVAT‐conditioned media from HFD female rats reduced mesenteric artery vasodilation in response to acetylcholine.

**FIGURE 5:**
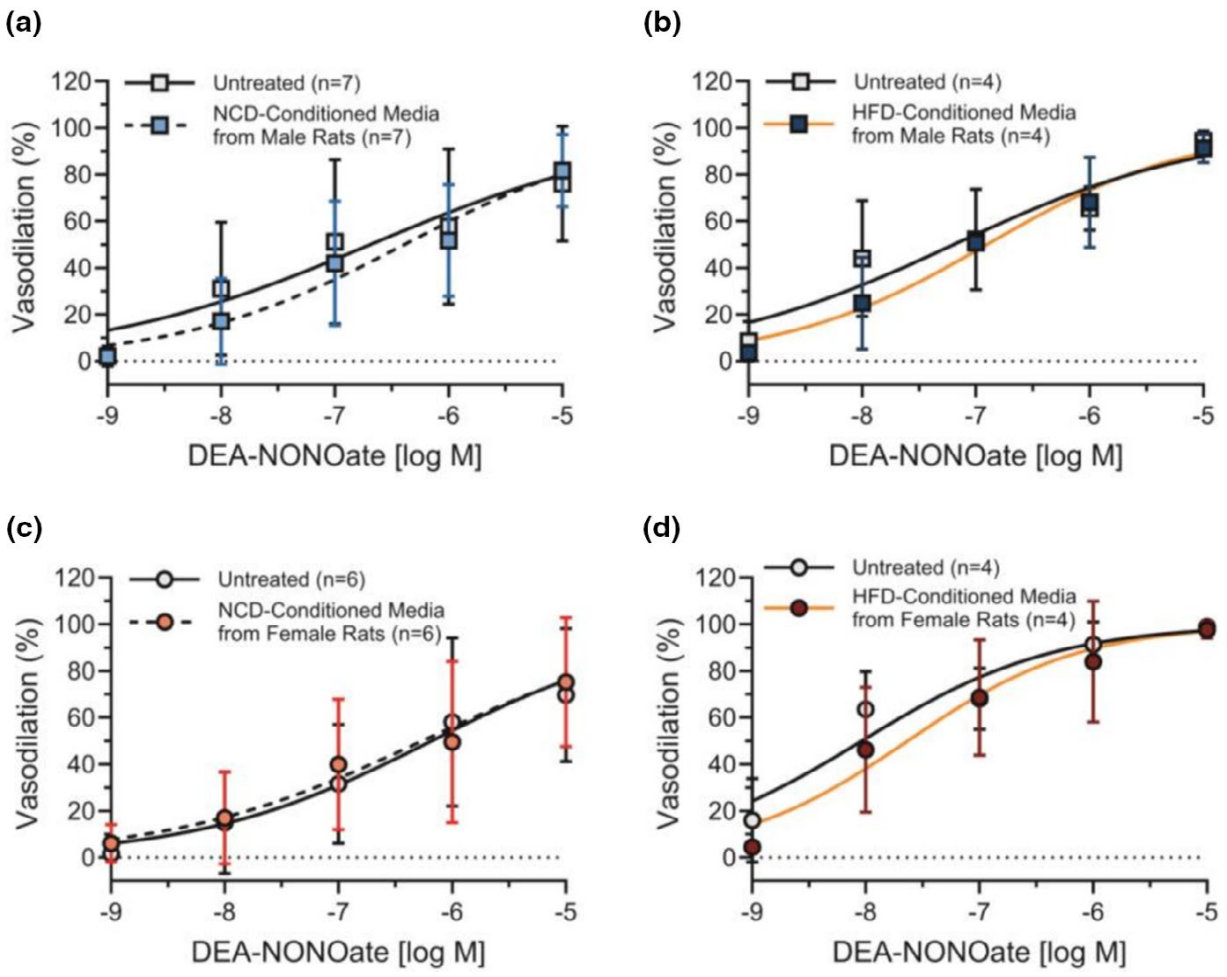
PVAT‐conditioned media from male and female rats did not impair smooth muscle cell relaxation. Pressure myography of mesenteric arteries undergoing DEA‐NONOate (NO donor) dose–response after 30‐min treatment with PSS (*n* = 4–7; untreated), or 10% conditioned media from NCD male rats (*n* = 7; a), HFD male rats (*n* = 3; b), NCD female rats (*n* = 6; c), and HFD female rats (*n* = 4; d). PVAT conditioned media effects on smooth muscle cell relaxation were tested on mesenteric arteries from sex and diet‐matched rats. Data are represented as mean ± standard deviation. Statistical significance was tested using a repeated measures ANOVA.

To determine if the HFD PVAT conditioned media acted on the endothelium, we then measured how PVAT‐conditioned media affected smooth muscle cell relaxation (endothelial‐independent vasodilation) by treating mesenteric arteries with the NO donor DEA‐NONOate. We did not observe significant differences in DEA‐NONOate‐induced vasodilation between untreated and PVAT‐conditioned media‐treated arteries from NCD or HCD male or female rats (Figure 5). Repeated‐measures ANOVA indicated a significant main effect of acetylcholine dose (*p* < 0.0001) only. These data show that female HFD PVAT‐induced changes in vasodilation are endothelial cell and not smooth muscle cell dependent.

### 3.4 PVAT‐conditioned media from HFD rats did not increase endothelial cell oxidative stress or impair endothelial eNOS activation or deactivation in vitro

**FIGURE 6:**
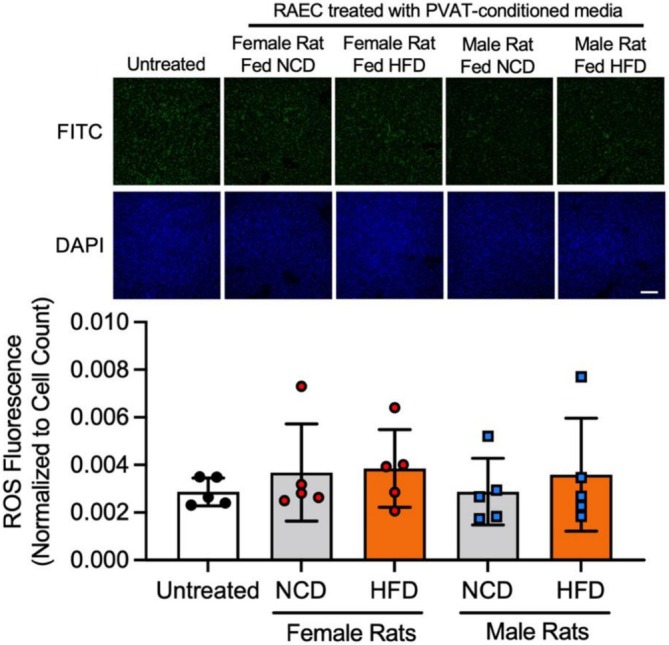
PVAT‐conditioned media from HFD rats did not increase endothelial oxidative stress. Representative fluorescent microscopy images and quantification of RAECs treated with PVAT‐conditioned media from male and female rats fed an NCD or HFD. Scale bar = 200 μm. All data were normalized to cell count. Data are represented as mean ± standard deviation using conditioned media from *n* = 5 rats. Statistical significance was determined using a one‐way ANOVA.

To investigate the mechanism underlying impaired vasodilation with PVAT‐conditioned media from HFD female rats, we studied how PVAT‐conditioned media affected endothelial NO bioavailability in vitro. We examined endothelial ROS as a marker for NO scavenging. RAEC treated with the positive control tBHP increased ROS fluorescence by 44% compared to untreated cells (*p* = 0.0079; Figure S7). However, PVAT‐conditioned media from male and female rats fed a NCD or HFD did not significantly affect ROS (Figure 6).

**FIGURE 7:**
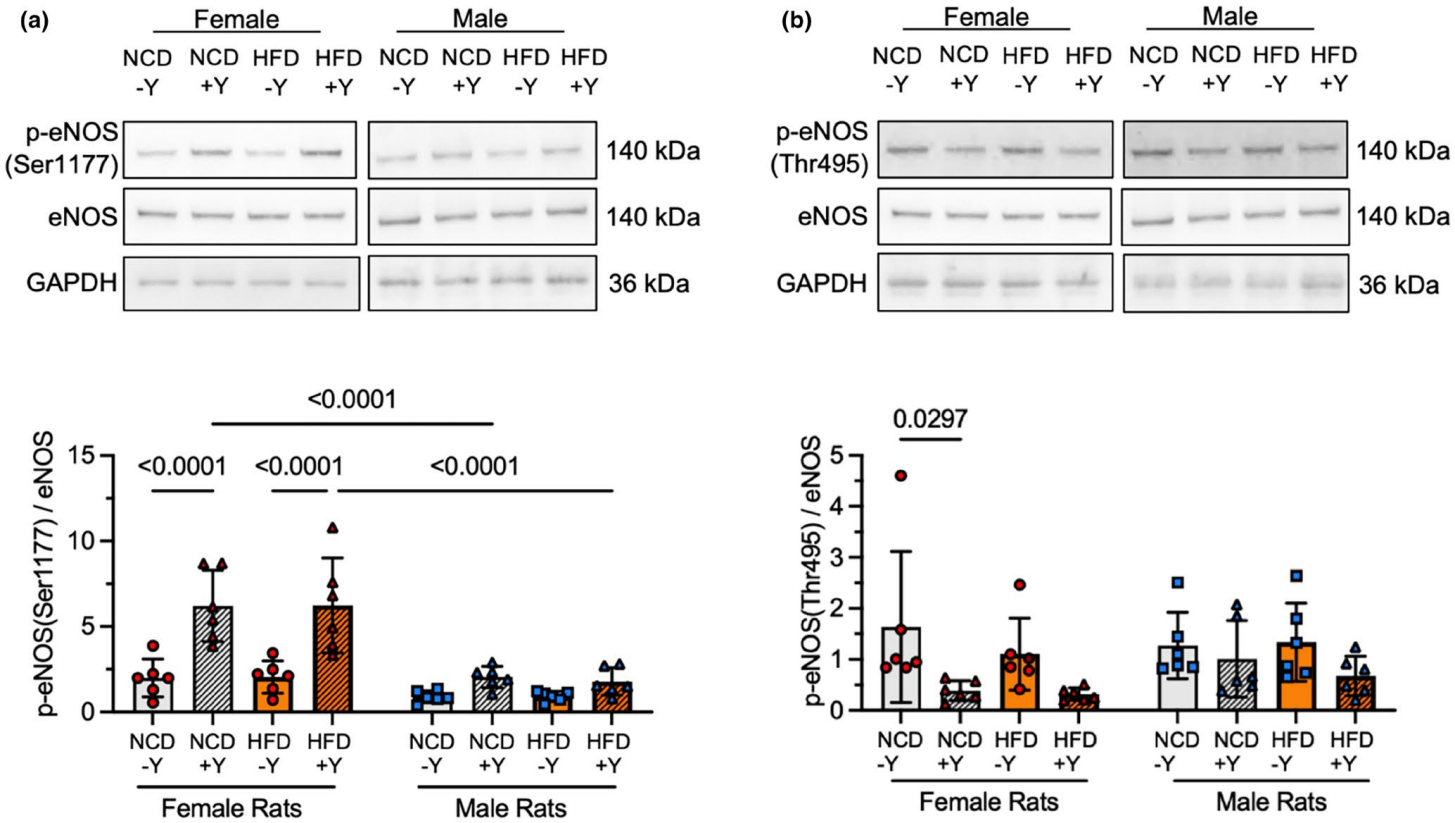
PVAT‐conditioned media from HFD rats did not impair eNOS activation or deactivation. Representative Western blots and quantification of eNOS (Ser1177; a), and eNOS (Thr495) phosphorylation (b) in RAEC pretreated with PVAT‐conditioned media from male and female rats fed an NCD or HFD and then treated with 2 μM Yoda1 (Y) for 30 min. All data normalized to GAPDH and one cell sample that was included on each Western blot. Data are represented as mean ± standard deviation from *n* = 6 rats per group. Statistical significance was determined using a two‐way ANOVA followed by Tukey multiple comparison test. The full Western blots are provided in the Data S1.

We then investigated how PVAT‐conditioned media affected phosphorylation of endothelial eNOS (Ser1177) as an indicator of eNOS activation. We treated some RAEC with Yoda1, an activator of the mechanosensitive ion channel Piezo1, as a positive control. Two‐way ANOVA indicated a significant main effect of sex (*p* < 0.0001), diet (*p* < 0.0001), and interaction between sex and diet (*p* = 0.0035). Yoda1 increased eNOS (Ser1177) phosphorylation by more than 200% in RAEC pretreated with female but not male PVAT‐conditioned media (*p* < 0.0001; Figure 7a). However, Yoda1‐induced eNOS (Ser1177) phosphorylation was not significantly different in RAEC pretreated with PVAT‐conditioned media from HFD rats compared to PVAT‐conditioned media from NCD rats, regardless of sex. Finally, we studied how PVAT‐conditioned media affected eNOS (Thr495) phosphorylation in Yoda1‐treated RAEC as an indicator for eNOS deactivation (Figure 7b). Two‐way ANOVA indicated no significant main effect of sex (*p* = 0.33), a significant main effect of diet (*p* = 0.0106), and no significant interaction between sex and diet (*p* = 0.43). RAEC pretreated with PVAT‐conditioned media from NCD female rats decreased eNOS (Thr495) phosphorylation by 76% (*p* = 0.0297) in response to Yoda1, while no other treatments had a significant effect. These results suggest that HFD PVAT‐conditioned media did not impact endothelial oxidative stress, eNOS activation, or eNOS deactivation.

## 4 DISCUSSION

The risk of CVD due to a HFD is higher in women than in men, highlighting the need to understand sex‐specific mechanisms that drive this difference. We therefore investigated how HFD‐induced changes in PVAT from male and female rats impacted vascular function. We found that some inflammatory markers were significantly higher with HFD in female but not male rats. HFD did not directly decrease acetylcholine‐induced vasodilation in male or female mesenteric arteries. However, endothelial‐dependent mesenteric artery sensitivity to acetylcholine‐induced vasodilation was impaired by PVAT from HFD female rats, which has not previously been reported. The mechanism underlying this effect remains unclear, since PVAT‐conditioned media from female rats did not affect oxidative stress or eNOS phosphorylation in rat endothelial cells in vitro. Finally, although PVAT‐conditioned media from male rats did not impair mesenteric artery vasodilation, it did reduce eNOS phosphorylation in response to Yoda1. Our results indicate that the female vasculature is more susceptible to HFD‐induced changes in PVAT, which may contribute to heightened cardiovascular risk in women who consume an HFD.

There are several possible reasons why PVAT‐conditioned media from HFD female rats could have reduced endothelial‐dependent vasodilation in mesenteric arteries but not increased ROS or decreased NO in rat endothelial cells in vitro. First, the rat endothelial cells that we used were a mix of aortic cells from male and female rats. The effects of female HFD PVAT could be specific to mesenteric and/or female endothelial cells. Second, HFD was shown to reduce PVAT NO production by decreasing eNOS (Ser1177) phosphorylation and shifting eNOS activity towards ROS production (Ma et al., 2010; Xia et al., 2016; Xia et al., 2017). Therefore, the PVAT‐conditioned media itself could have contained stable derivatives of NO or ROS, which then impacted endothelial‐dependent vasodilation directly instead of via endothelial eNOS. Additionally, hyperpolarization contributes to vasodilation in resistance arteries by increasing endothelial potassium efflux, thereby hyperpolarizing and relaxing vascular smooth muscle cells. However, we did not evaluate endothelium‐dependent hyperpolarization because our goal was to specifically investigate how PVAT affects pathways that regulate NO bioavailability. Finally, numerous studies showed that PVAT from HFD rodents increases smooth muscle cell contraction to phenylephrine (Aghamohammadzadeh et al., 2016; Aoqui et al., 2014; da Costa et al., 2017; Han et al., 2018; Ma et al., 2010; Victorio et al., 2021). Thus, the PVAT‐conditioned media also could have contained vasoconstrictors, such as endothelin, that inhibited vasodilation by enhancing contraction (Nishiyama et al., 2017). Additional research is needed to clarify how PVAT from HFD female rats impairs endothelial‐dependent vasodilation.

In our study, endothelial‐dependent vasodilation was not impaired by PVAT from HFD male rats. This result was unexpected, since multiple reports in the literature showed that PVAT from HFD male rats impairs acetylcholine‐induced vasodilation (Aoqui et al., 2014; Horimatsu et al., 2018; Ma et al., 2010; Sousa et al., 2019; Xia et al., 2016). Our HFD may not have disrupted PVAT function in male rats because the diet only caused a modest increase in body and PVAT weight. In rodent models of HFD‐induced obesity, a body weight increase of 10%–25% compared to age‐matched rats fed a NCD is classified as moderately obese (Hariri & Thibault, 2010). In most studies of PVAT effects on vascular function, animal body weight increased 20%–75% with a HFD (Aghamohammadzadeh et al., 2016; Almabrouk et al., 2018; DeVallance et al., 2018; Ma et al., 2010; Tran et al., 2023; Victorio et al., 2021; Xia et al., 2016; Xia et al., 2017). Sousa et al. demonstrated that male C57BL/6JUnib mice fed a HFD for 16 weeks experienced a 67.9% increase in body weight and a 275% increase in thoracic PVAT (DeVallance et al., 2018), while Ma et al. showed that male Wistar rats fed a HFD for 24 weeks increased body weight by 43% and PVAT by 41.5% (Ma et al., 2010). In our study, the HFD male rats showed an 8.5% increase in body weight compared to NCD rats, while the PVAT weight increased by 15.2%. Therefore, HFD‐induced vascular dysfunction in male rats may require substantial weight gain before impairments occur.

HFD also did not impact vasodilatory PVAT adipokines such as adiponectin in our male or female rats. Adiponectin independently stimulates both NO‐dependent vasodilation and smooth muscle cell relaxation, and HFD has been shown to decrease PVAT anti‐contractility effects by reducing adiponectin secretion (Cheng et al., 2007; Hong et al., 2016; Schmid et al., 2011). In previous studies using male rodents, PVAT adiponectin mRNA decreased in C57Bl6/J mice fed a HFD for 20 weeks (Xia et al., 2016), and adiponectin protein was reduced in PVAT from C57Bl6/J and Sv129 mice fed a HFD for 8 weeks (Almabrouk et al., 2018; Aoqui et al., 2014). However, our data did not show changes in PVAT adiponectin, indicating that adiponectin was not the cause of the diminished vasodilation observed due to PVAT from HFD female rats. Together, these findings suggest that reduced adiponectin secretion by PVAT is a hallmark of obesity rather than a direct consequence of HFD feeding.

HFD did increase PVAT inflammation, with larger pro‐inflammatory changes in PVAT from female compared to male rats. Similarly, male C57Bl/6 mice fed a HFD for 8–20 weeks had elevated PVAT inflammation, indicated by increased gene expression of CD68 macrophage and CD45 lymphocyte markers and inflammatory cytokines tumor necrosis factor α (TNFα) and monocyte chemoattractant protein‐1 (MCP‐1) (Aoqui et al., 2014; Horimatsu et al., 2018; Xia et al., 2016). Inflammatory cytokines like TNFα impair anti‐contractility by exacerbating oxidative stress (Aghamohammadzadeh et al., 2016; da Costa et al., 2017; DeVallance et al., 2018; Wang et al., 2012). For example, HFD male mice lacking TNFα receptors exhibited reduced ROS production and phenylephrine‐induced aortic constriction. Antioxidants also reduced phenylephrine‐induced aortic constriction in PVAT‐intact mesenteric arteries from HFD male rats (Aghamohammadzadeh et al., 2016; da Costa et al., 2017). In our study, however, PVAT‐conditioned media from male and female rats did not increase ROS in endothelial cells, suggesting that the observed impairment in vasodilation was not caused by inflammation‐induced oxidative stress.

We observed several interesting differences between male and female rat vasculature, independent of diet. Mesenteric arteries from female rats showed greater acetylcholine‐induced vasodilation than those from male rats. These results are consistent with White et al., who also used pressure myography to show that mesenteric arteries from female Sprague–Dawley rats experienced greater acetylcholine‐induced vasodilation than mesenteric arteries from male rats (White et al., 2000). We further observed that endothelial cells treated with PVAT‐conditioned media from female rats exhibited greater eNOS (Ser1177) phosphorylation and greater eNOS (Thr495) dephosphorylation compared to cells treated with male PVAT‐conditioned media. These data support the idea that both female arteries and PVAT have higher vasodilatory properties as compared to male arteries and PVAT.

We also observed sex differences in PVAT leptin gene expression. Leptin protects against endothelial dysfunction in male but not female mice (Mellott & Faulkner, 2023), with exogenous leptin improving acetylcholine‐induced aortic relaxation in male mice while impairing it in female mice (Faulkner et al., 2019; Huby et al., 2015; Huby et al., 2016). In our study, PVAT from male rats had greater leptin gene expression than PVAT from female rats, regardless of diet. This suggests that male rats may have additional pro‐vasodilatory signaling than female rats. Conversely, HFD female rats exhibited greater circulating leptin than male rats, likely reflecting leptin release from other adipose depots that may impair vascular function in vivo. While our findings suggest sex differences in local and circulating leptin may promote vasodilation in male rats and impair it in female rats, leptin levels in PVAT‐conditioned media did not differ by sex or diet. Thus, other mechanisms likely contributed to the impaired vasodilation in female rat mesenteric arteries treated with HFD PVAT‐conditioned media.

This study has limitations that constrain our ability to fully elucidate how PVAT‐conditioned media affects vascular function. Our in vitro experiments used pooled male and female endothelial cells, since our objective was to study sex differences in the PVAT. However, this may have obscured some sex‐specific pathways since PVAT‐conditioned media from female rats could uniquely affect female endothelial cells. The observed effects may also be unique to PVAT from the thoracic aorta, as Victorio et al. found that mesenteric PVAT from female rats fed a HFD for 20 weeks did not impair mesenteric artery vasorelaxation (Victorio et al., 2021). Finally, we tested the effects of conditioned media from thoracic PVAT on mesenteric artery vasodilation due to the previously described experimental constraints. While pressure myography enabled us to study the effects of thoracic PVAT on intact, pressurized arteries, we had to use previously frozen PVAT‐conditioned media rather than fresh conditioned media or intact PVAT. Freezing PVAT‐conditioned media prior could have impacted protein structure or stability, masking some of the conditioned media effects. These limitations highlight the need for future studies to explore sex‐specific mechanisms that drive HFD‐induced vascular dysfunction.

This study highlights sex‐specific differences in how diet modulates PVAT function and its paracrine effects on vascular health. The observed early inflammatory and metabolic shifts in PVAT from female rats indicate higher female sensitivity to HFD. These effects of HFD on PVAT and subsequent vascular dysfunction occur in the absence of significant adipose tissue gains, suggesting that women may experience detrimental effects of a HFD without becoming obese. Thus, sex should be considered as an essential biological variable when investigating the interplay among diet, adipose tissue, and vascular function and when providing preventative CVD care. These insights should guide the development of sex‐specific diagnostic and therapeutic strategies aimed at mitigating diet‐induced cardiovascular complications in both men and women.

## 5 CONCLUSION

Our study demonstrates that HFD promotes distinct sex‐dependent molecular changes in the thoracic PVAT that impair endothelial function despite modest weight gain. Both male and female rats fed a HFD did not significantly increase body weight, PVAT weight, or PVAT adipokines compared to rats on a NCD. However, HFD female rats exhibited a more pronounced pro‐inflammatory response in PVAT. Additionally, PVAT from female rats fed a HFD impaired endothelium‐dependent vasodilation in mesenteric arteries. These findings emphasize the importance of exploring sex‐specific mechanisms in CVD, particularly concerning diet, and suggest that targeting PVAT may offer therapeutic strategies to prevent endothelial dysfunction and slow CVD progression.

## AUTHOR CONTRIBUTIONS

G.S.S. and R.M.S. wrote the introduction, R.M.S. and G.S.S wrote the methods, and G.S.S. wrote the abstract, results, discussion, and conclusions sections. R.M.S. performed pressure myography experiments, RT‐qPCR, and Western blots in PVAT. G.S.S. performed pressure myography experiments and ROS assays in endothelial cells. C.M.W. and D.T. performed Western blot in endothelial cells. C.M.W., D.T., N.R., and A.B. cultured, collected, and stored PVAT‐conditioned media. G.S.S., R.M.S., and A.M.C. designed all experiments. A.M.C. oversaw all experiments and the preparation of the figures and manuscript. All authors contributed to the article and approved the submitted version.

## FUNDING INFORMATION

Funding: University of Maryland Presidential Postdoctoral Fellowship: Gurneet Singh Sangha, N/A; American Heart Association Postdoctoral Fellowship: Gurneet Singh Sangha, 916512; American Heart Association Postdoctoral Fellowship: Ryan M Sapp, 915916; National Institutes of Health Postdoctoral Institutional Training Grant: Ryan M Sapp, 5T32HL007698‐26; National Science Foundation (NSF) Graduate Research Fellowship Program: Callie M Weber, DGE 1840340; HHS | National Institutes of Health (NIH): Alisa Morss Clyne, R21EB028466; HHS | National Institutes of Health (NIH): Alisa Morss Clyne, R01HL140239; HHS | National Institutes of Health (NIH): Alisa Morss Clyne, R01HL165193; National Science Foundation (NSF): Alisa Morss Clyne, CMMI 1916814 Funding support was provided to G.S.S. through the University of Maryland Presidential Postdoctoral Fellowship and the American Heart Association Postdoctoral Fellowship (916512), R.M.S. by a National Institutes of Health Postdoctoral Institutional Training Grant (5T32HL007698‐26) and an American Heart Association Postdoctoral Fellowship (915916), C.W. through the National Science Foundation Graduate Research Fellowship Program (DGE 1840340), and A.M.C through the National Science Foundation (CMMI 1916814) and National Institutes of Health grants (R21EB028466, R01HL140239, R01HL165193).

## CONFLICT OF INTEREST STATEMENT

The authors declare that they have no competing interests.

## ETHICS STATEMENT

All animal experiments were approved by the Institutional Animal Care and Use Committee (IACUC) of the University of Maryland (Project ID#: 1643983‐8). Rat aortic endothelial cells were obtained from commercial sources and used in accordance with the suppliers' ethical guidelines. Human subjects were not used in this study.

## Supporting information


Data S1:

Data S2:

## Data Availability

The datasets used and analyzed in this study are available from the corresponding author on reasonable request.
