# Supplementary material for: Perivascular adipose tissue from female rats fed a high‐fat diet impaired mesenteric artery vasodilation
Source: Physiol Rep. 2026 Jan 28;14(2):e70746. doi: 10.14814/phy2.70746 (PMC12849210; doi:10.14814/phy2.70746)
Supplement: Supplementary file 1 — Data S1: [file PHY2-14-e70746-s001.docx]

**Supplementary Figures**


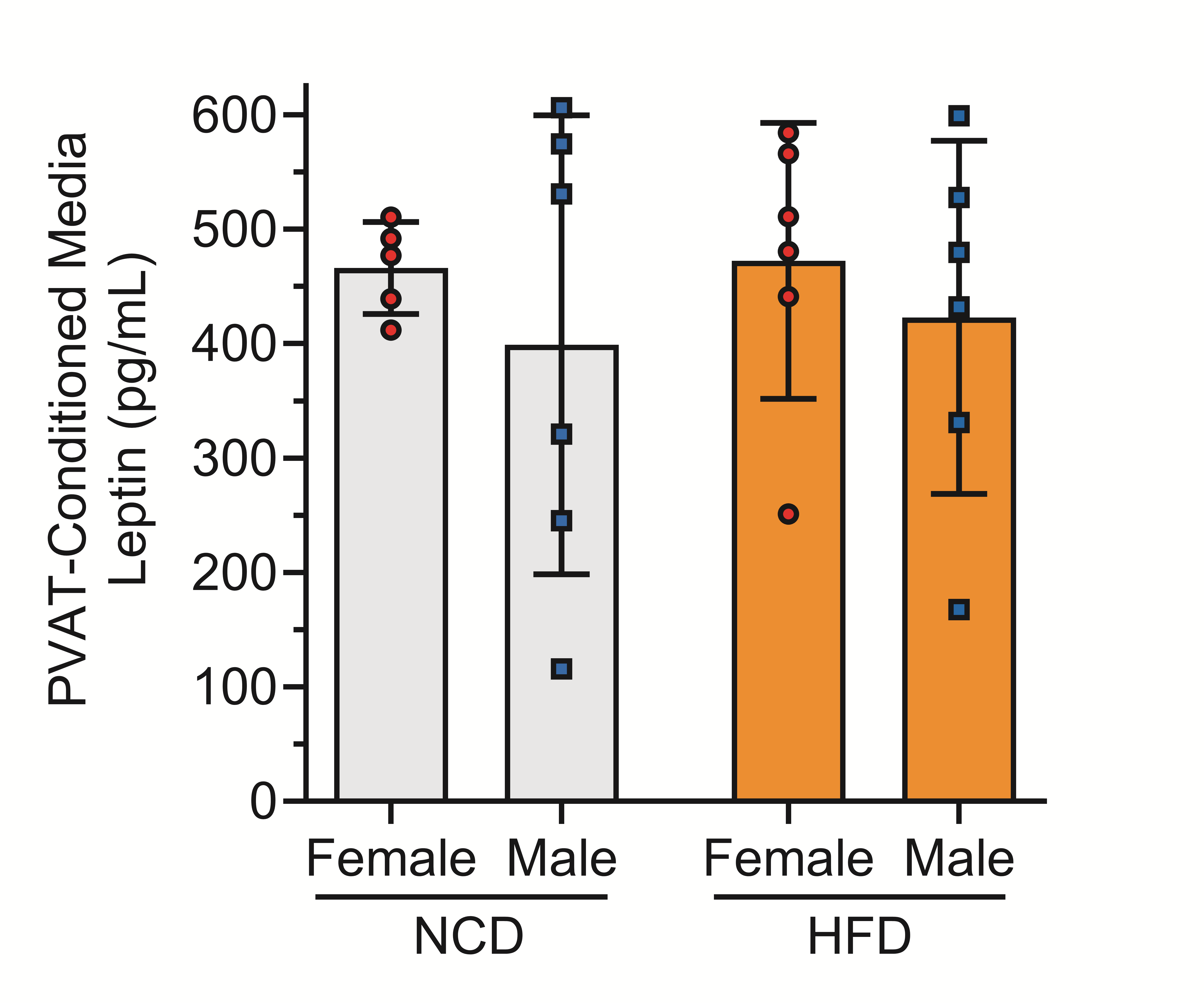


**Supplementary Figure 1. Neither sex nor HFD increased leptin concentration in PVAT-conditioned media.** Leptin concentration in PVAT conditioned media from NCD female rats (n=5), HFD female rats (n=6), NCD male rats (n=6), and HFD male rats (n=6), as measured by ELISA. Data are represented as mean ± standard deviation. Statistical significance was determined using a two-way ANOVA.





**Supplementary Figure 2. HFD increased plasma leptin in female but not male rats.** Plasma leptin in NCD female rats (n=5), HFD female rats (n=5), NCD male rats (n=6), and HFD male rats (n=6), as measured by ELISA. Data are represented as mean ± standard deviation. Statistical significance was determined using a two-way ANOVA followed by Fisher’s LSD test.


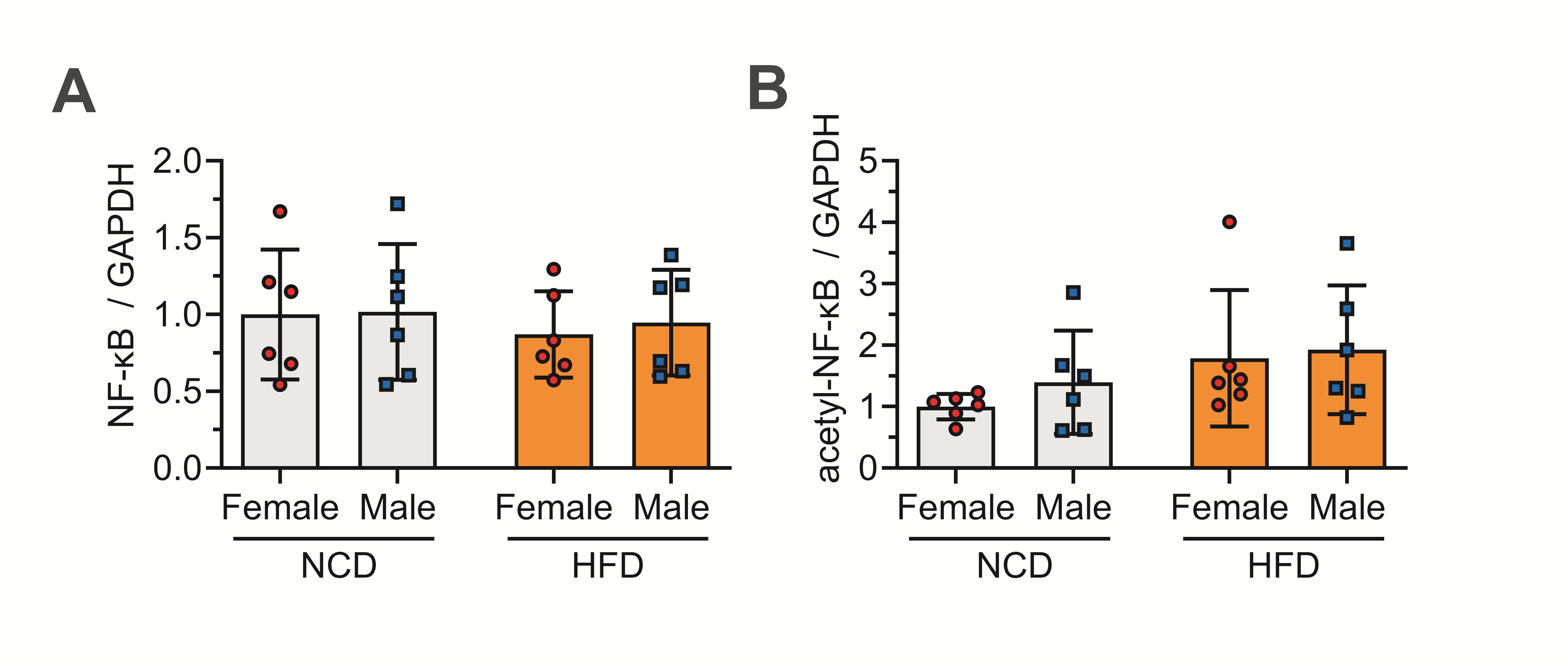


**Supplementary Figure 3. PVAT total and acetyl-NF-κB did not change with sex or diet.** Quantified Western blot data for NF-κB (A) and acetyl-NF-κB (B) in PVAT from NCD and HFD male and female rats. n=6 rats per group. Data are represented as mean ± standard deviation. Statistical significance was determined using a two-way ANOVA.


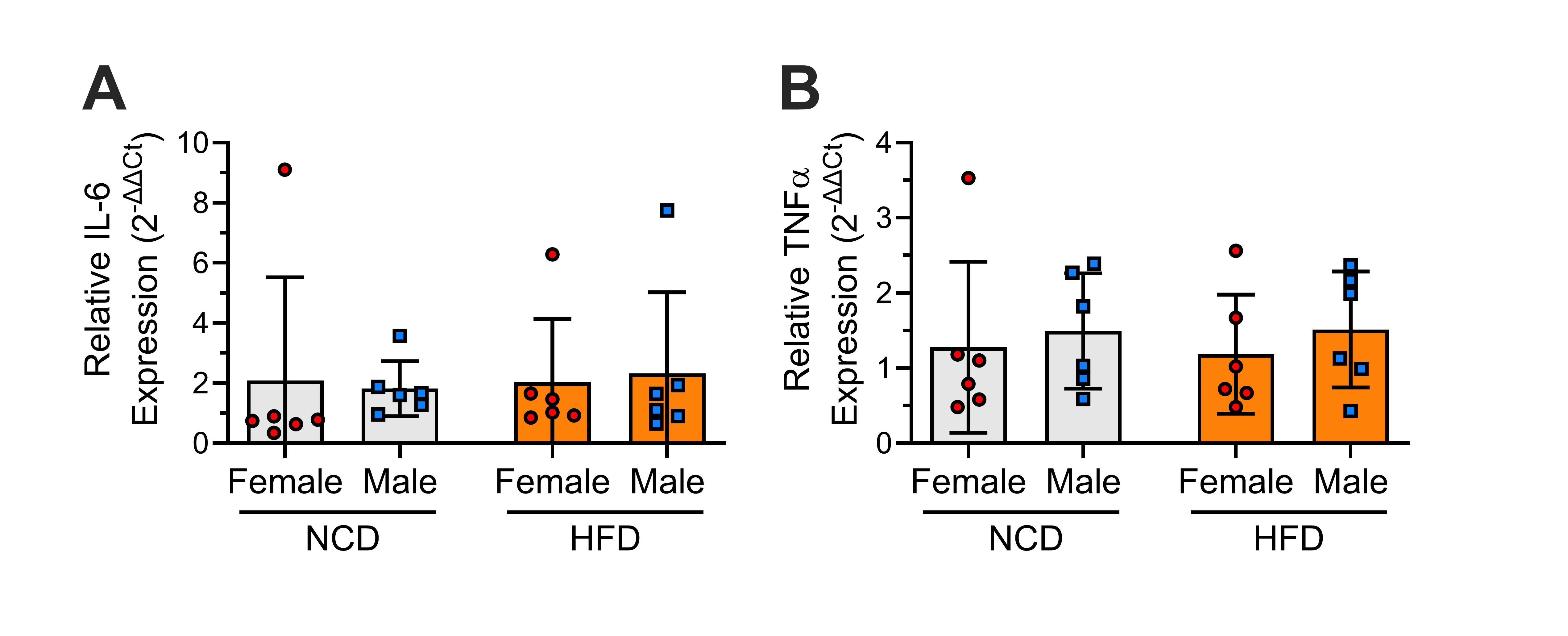


**Supplementary Figure 4: PVAT from HFD rats did not have higher inflammatory cytokine gene expression than PVAT from NCD rats.** Relative gene expression of IL-6 (A) and TNFα (B) in PVAT from NCD and HFD female and male rats. n=6 rats per group. Data represented as mean ± standard deviation. Statistical significance testing was determined using a two-way ANOVA.


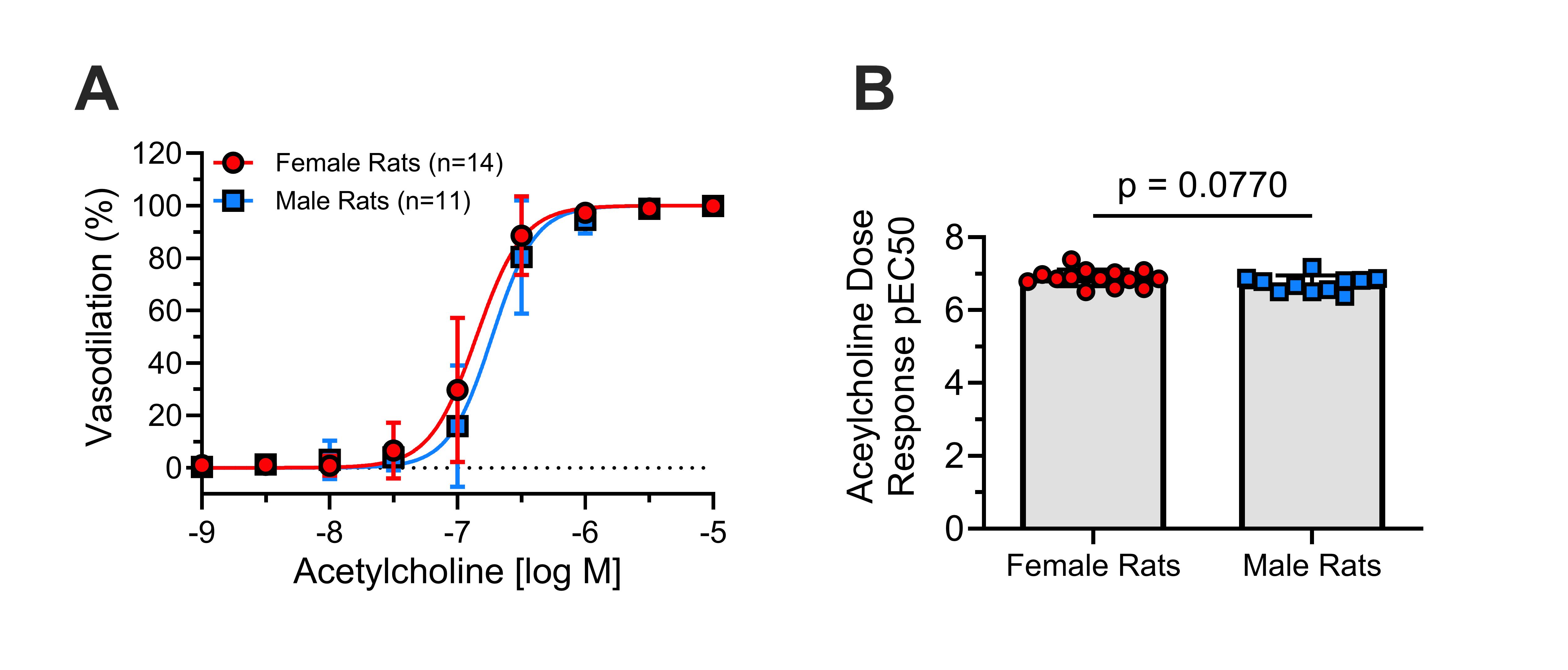


**Supplementary Figure 5. Female and male rat mesenteric arteries dilated similarly in response to acetylcholine.** Pressure myography (A) and pEC50 (B) for male and female mesenteric arteries undergoing acetylcholine dose-response. Data were pooled from all female rats (NCD and HFD; red circles) and all male rats (NCD and HFD; blue squares). Mesenteric arteries were not treated with PVAT conditioned media. Data are represented as mean ± standard deviation. Statistical significance for B was determined using a Mann-Whitney test.


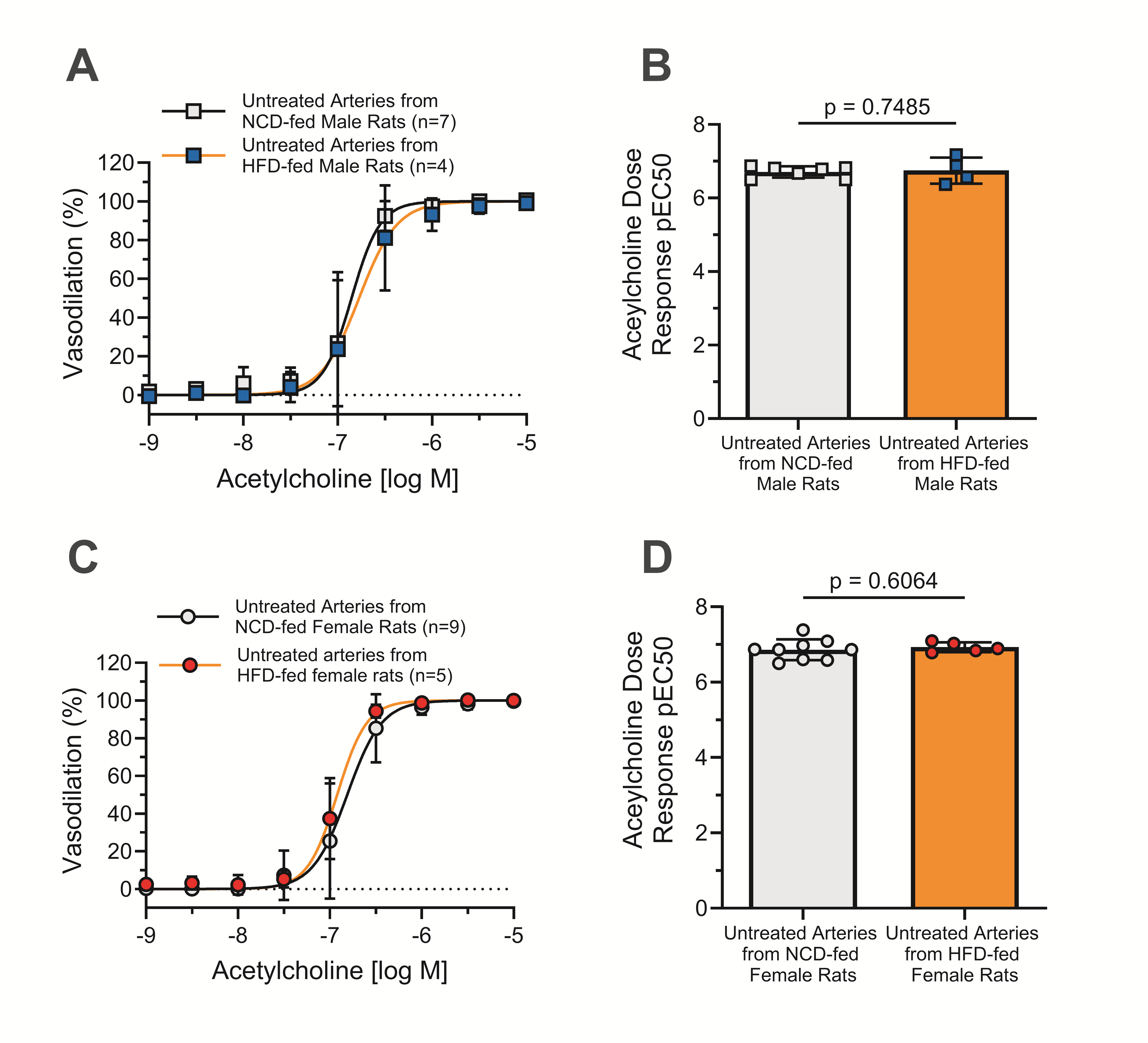


**Supplementary Figure 6. HFD did not impair acetylcholine-induced vasodilation in untreated rat mesenteric arteries.** Pressure myography (A,C) and pEC50 (B,D) for mesenteric arteries undergoing acetylcholine dose-response in male (A,B) and female (C,D) rats without conditioned media treatment. Data are represented as mean ± standard deviation. Statistical significance for B and D was determined using a Mann-Whitney test.


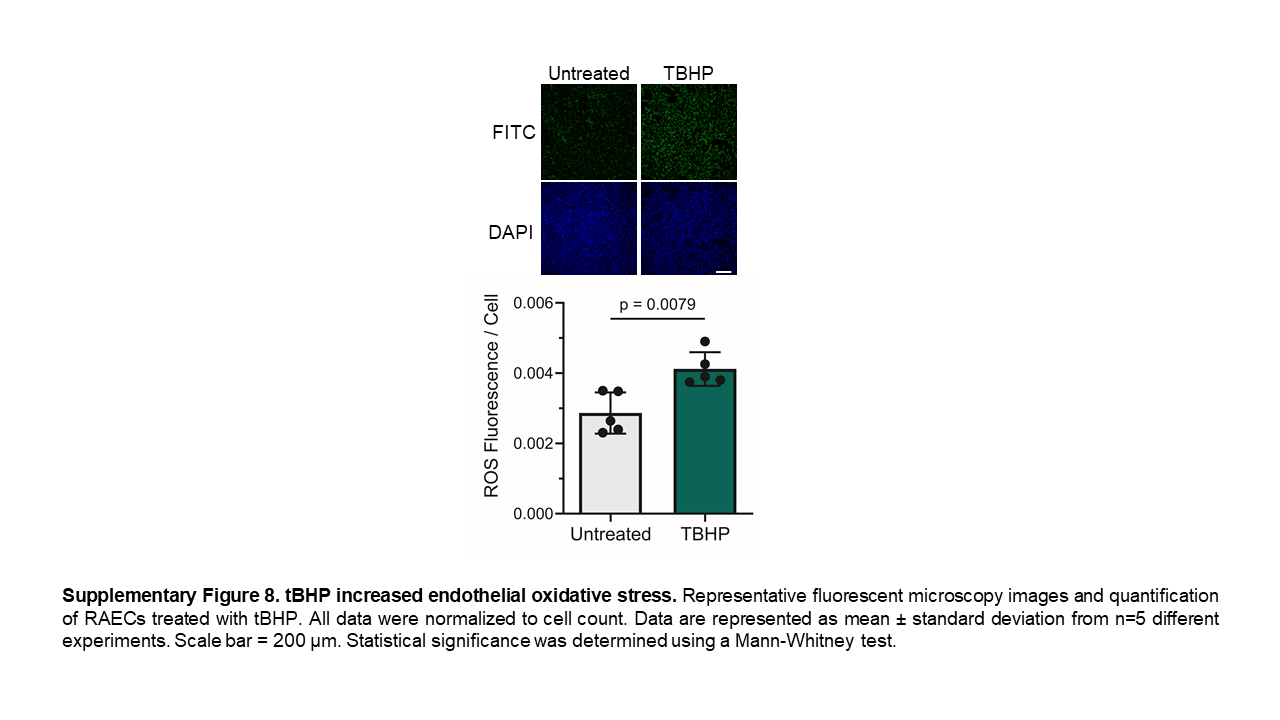


**Supplementary Figure 7. tBHP increased endothelial oxidative stress.** Representative fluorescent microscopy images and quantification of RAECs treated with tBHP. All data were normalized to cell count. Data are represented as mean ± standard deviation from n=5 experiments. Scale bar = 200 µm. Statistical significance was determined using a Mann-Whitney test.
