# Supplementary material for: Perivascular adipose tissue from female rats fed a high‐fat diet impaired mesenteric artery vasodilation
Source: Physiol Rep. 2026 Jan 28;14(2):e70746. doi: 10.14814/phy2.70746 (PMC12849210; doi:10.14814/phy2.70746)
Supplement: Supplementary file 2 — Data S2: [file PHY2-14-e70746-s002.pdf]

Figure 3
Western Blot Data

Images highlighted in red boxes were used as representative images in figures.

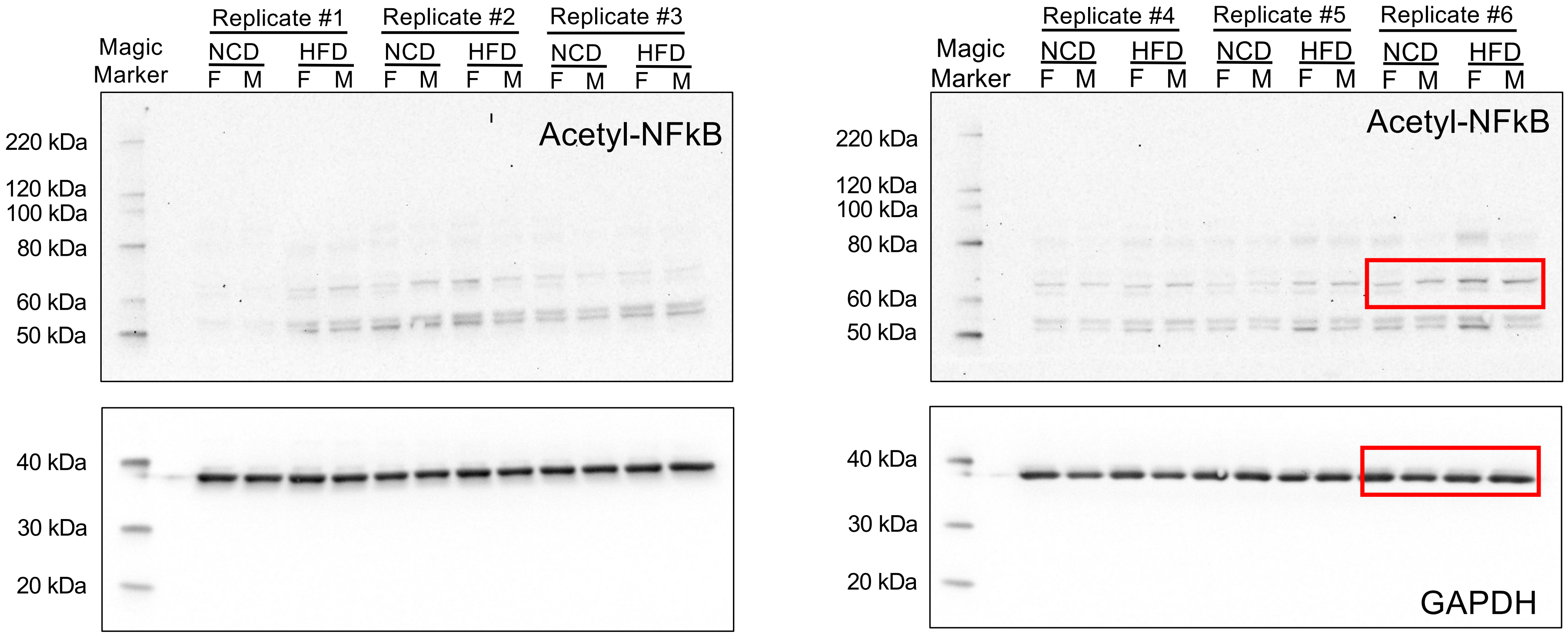
Replicate #1
Replicate #2
Replicate #3
Magic Marker
NCD
HFD
F M
Acetyl-NFkB
220 kDa
120 kDa
100 kDa
80 kDa
60 kDa
50 kDa
40 kDa
30 kDa
20 kDa
Replicate #4
Replicate #5
Replicate #6
GAPDH

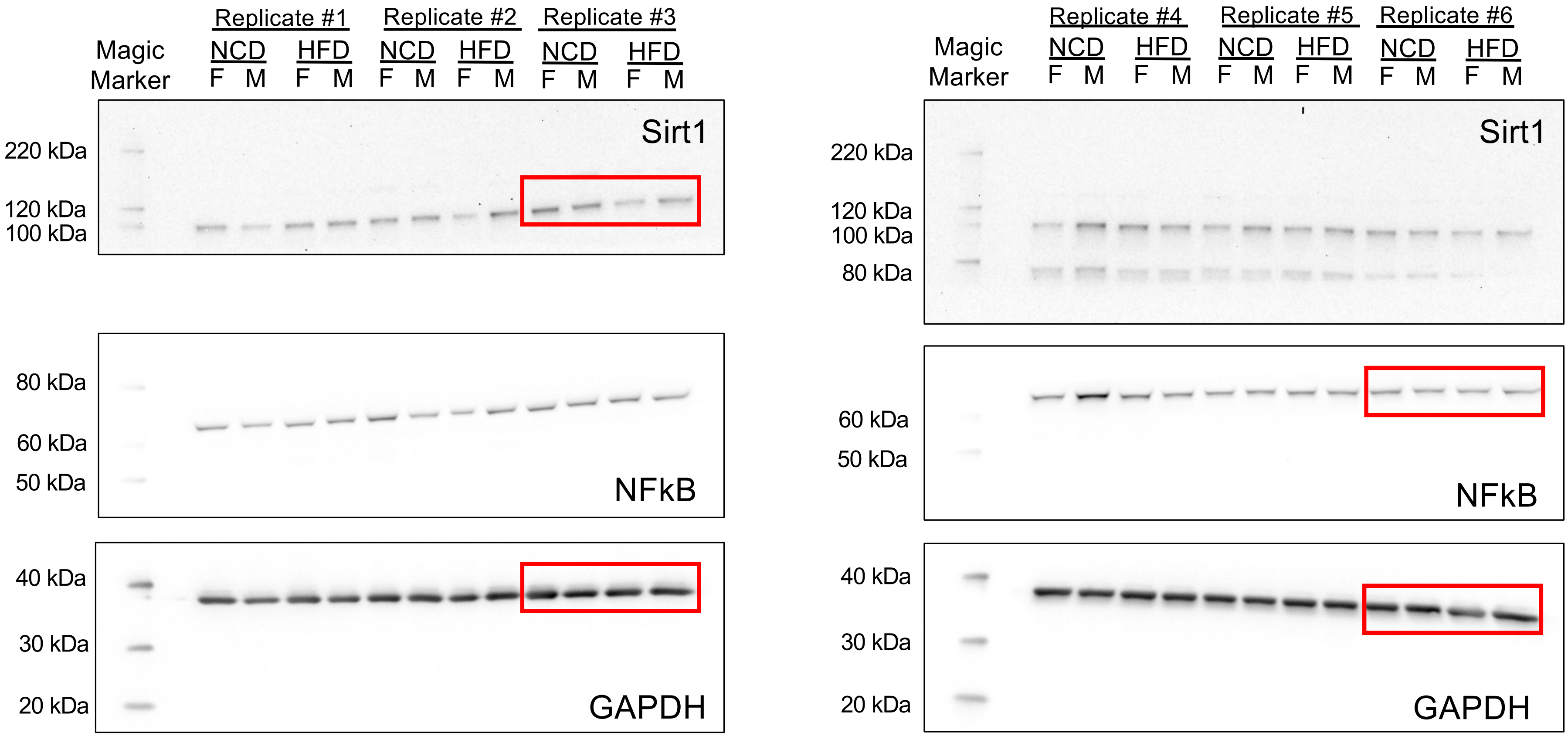
Replicate #1
Replicate #2
Replicate #3
Magic Marker
NCD HFD NCD HFD NCD HFD
F M F M F M F M F M F M
220 kDa
120 kDa
100 kDa
Sirt1
80 kDa
60 kDa
50 kDa
NFkB
40 kDa
30 kDa
20 kDa
GAPDH
Replicate #4
Replicate #5
Replicate #6
Magic Marker
NCD HFD NCD HFD NCD HFD
F M F M F M F M F M F M
220 kDa
120 kDa
100 kDa
80 kDa
Sirt1
60 kDa
50 kDa
NFkB
40 kDa
30 kDa
20 kDa
GAPDH

Figure 7
Female Rat
Western Blot Data

Images highlighted in red boxes were used as representative images in figures.
‘N. Lysate’ denotes lysate used to normalize and pool data together from multiple Western blots.

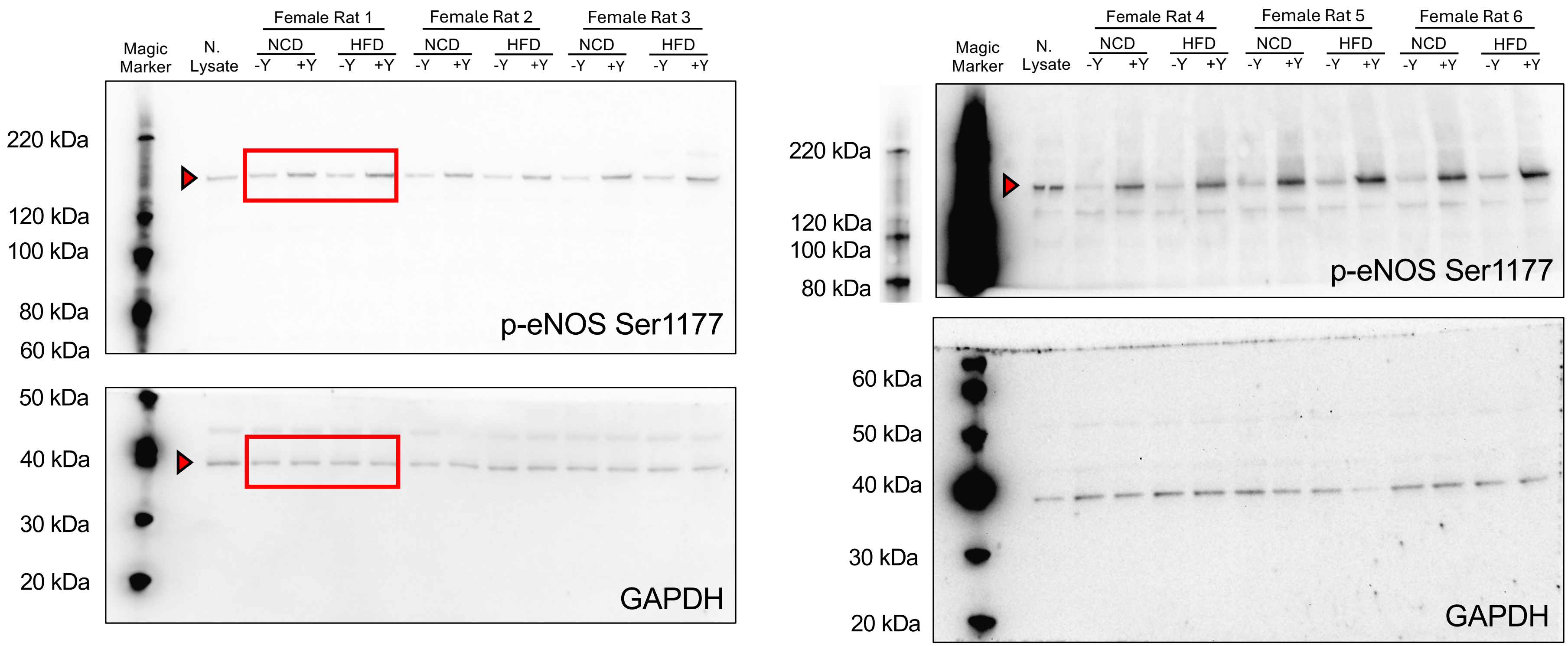

Female Rat 1
Female Rat 2
Female Rat 3
Magic Marker
N. Lysate
NCD
HFD
-Y
+Y
220 kDa
120 kDa
100 kDa
80 kDa
60 kDa
50 kDa
40 kDa
30 kDa
20 kDa
p-eNOS Ser1177
GAPDH
Female Rat 4
Female Rat 5
Female Rat 6

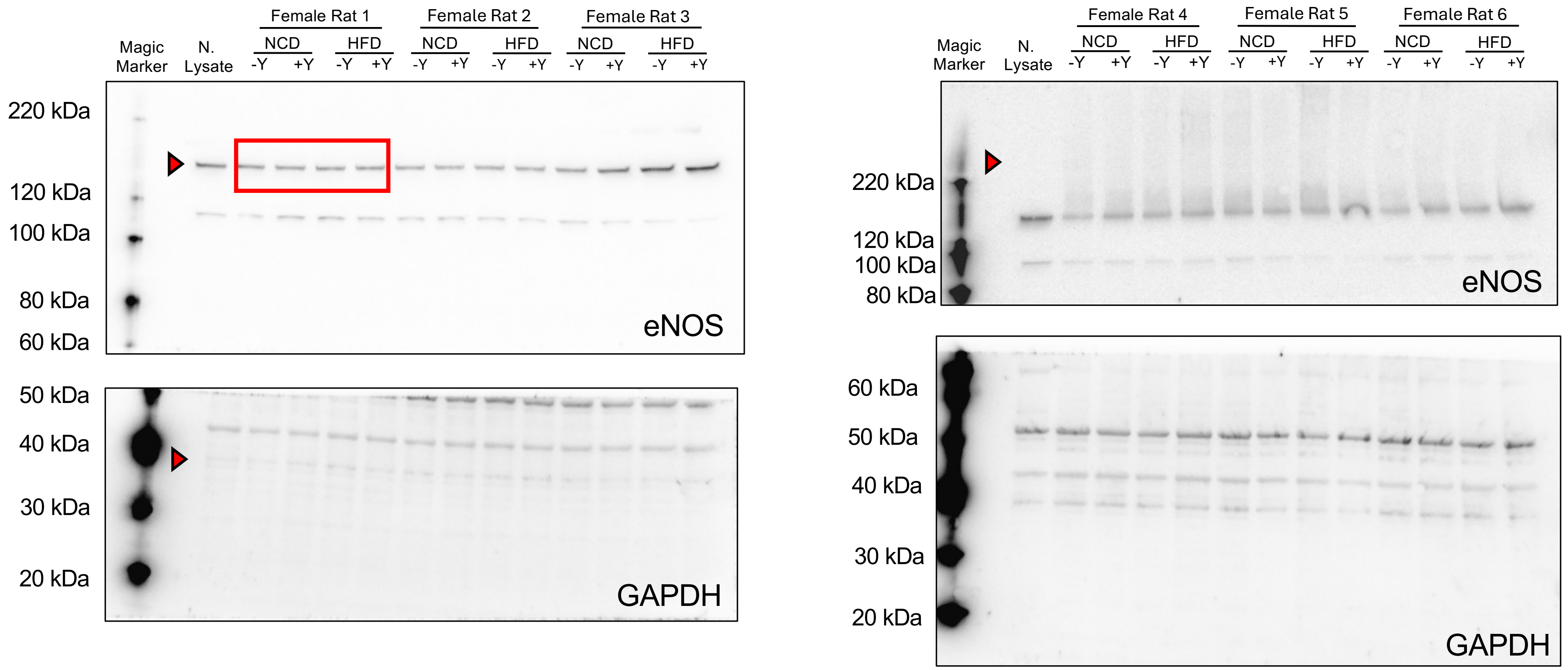

Female Rat 1
Female Rat 2
Female Rat 3
Magic Marker
N. Lysate
NCD
HFD
-Y
+Y
220 kDa
120 kDa
100 kDa
80 kDa
60 kDa
eNOS
50 kDa
40 kDa
30 kDa
20 kDa
GAPDH
Female Rat 4
Female Rat 5
Female Rat 6

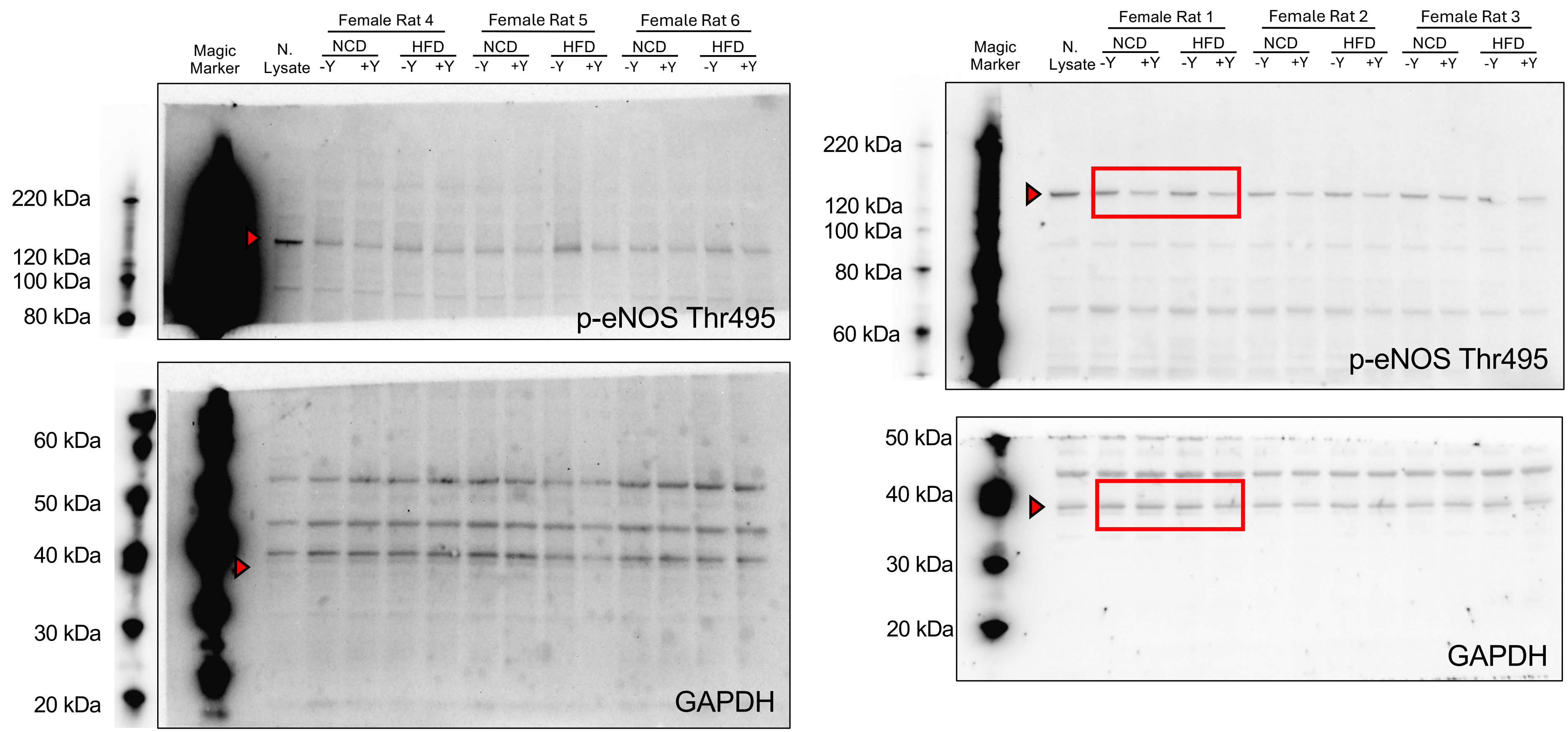
Female Rat 4
Female Rat 5
Female Rat 6
Magic Marker
N. Lysate
NCD
HFD
-Y
+Y
220 kDa
120 kDa
100 kDa
80 kDa
p-eNOS Thr495
60 kDa
50 kDa
40 kDa
30 kDa
20 kDa
GAPDH
Female Rat 1
Female Rat 2
Female Rat 3

Figure 7
Male Rat
Western Blot Data

Images highlighted in red boxes were used as representative images in figures.
‘N. Lysate’ denotes lysate used to normalize and pool data together from multiple Western blots.

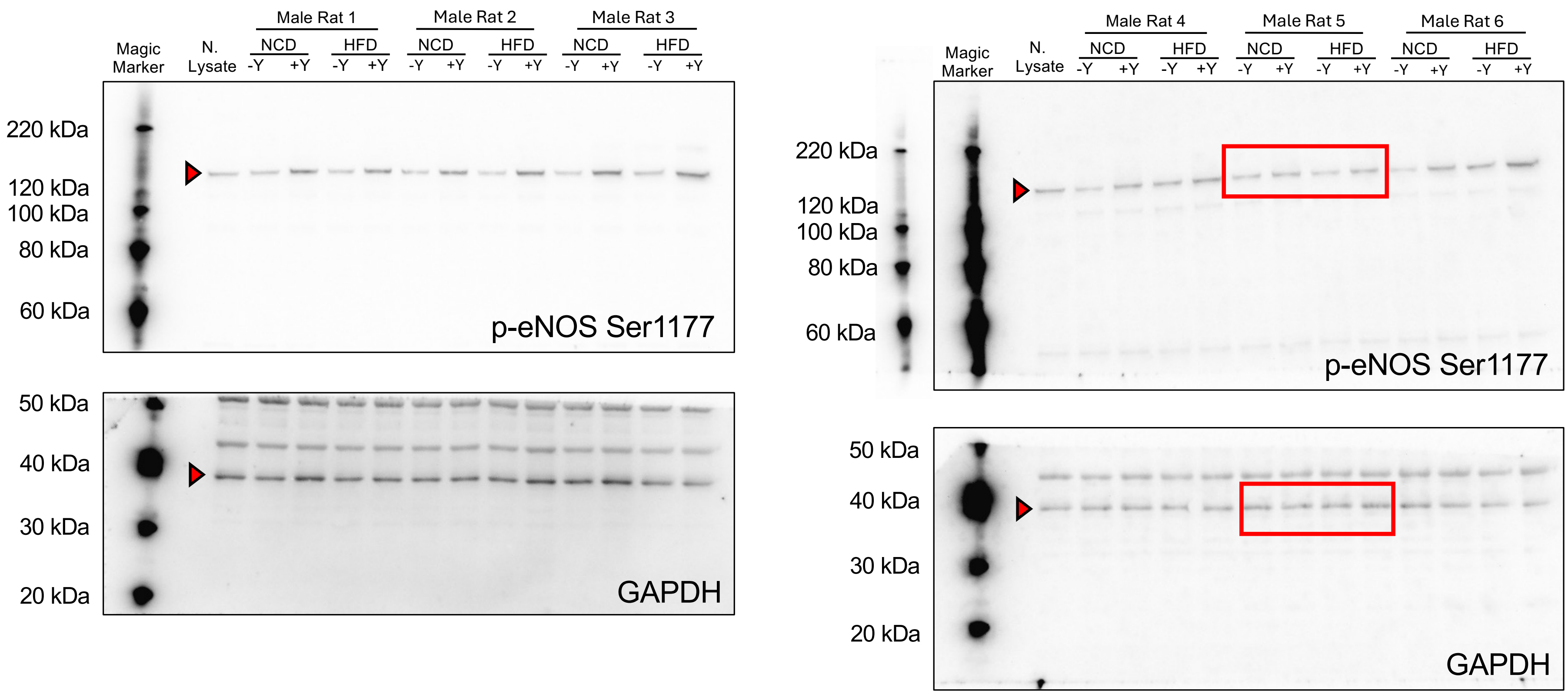

Magic Marker
N. Lysate
Male Rat 1
Male Rat 2
Male Rat 3
NCD
HFD
-Y
+Y
220 kDa
120 kDa
100 kDa
80 kDa
60 kDa
p-eNOS Ser1177
50 kDa
40 kDa
30 kDa
20 kDa
GAPDH
Male Rat 4
Male Rat 5
Male Rat 6

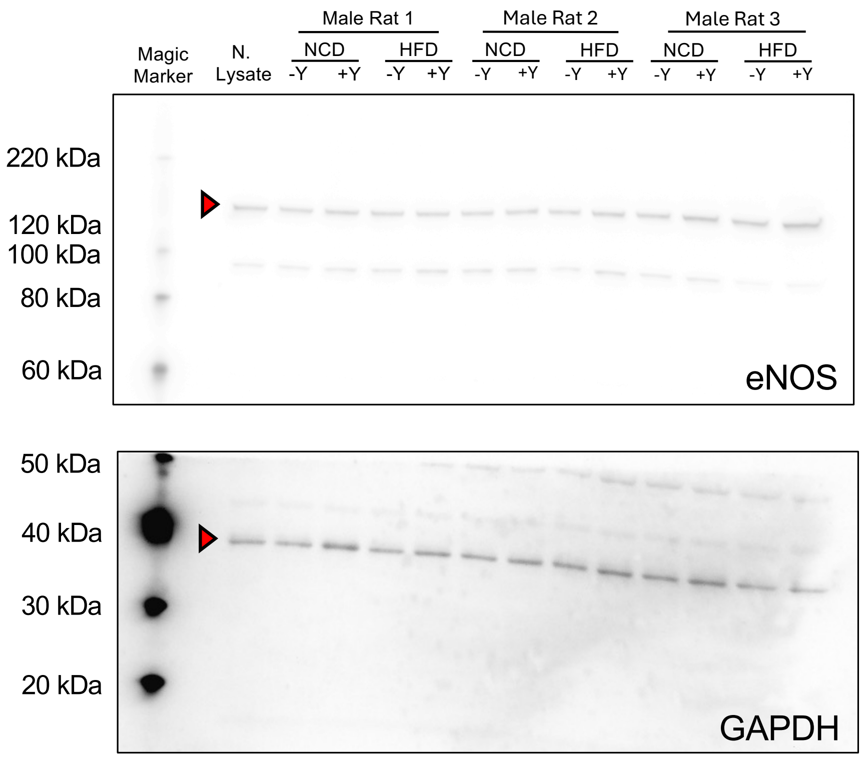
Magic Marker
N. Lysate
Male Rat 1
Male Rat 2
Male Rat 3
NCD
HFD
-Y
+Y
220 kDa
120 kDa
100 kDa
80 kDa
60 kDa
eNOS
50 kDa
40 kDa
30 kDa
20 kDa
GAPDH

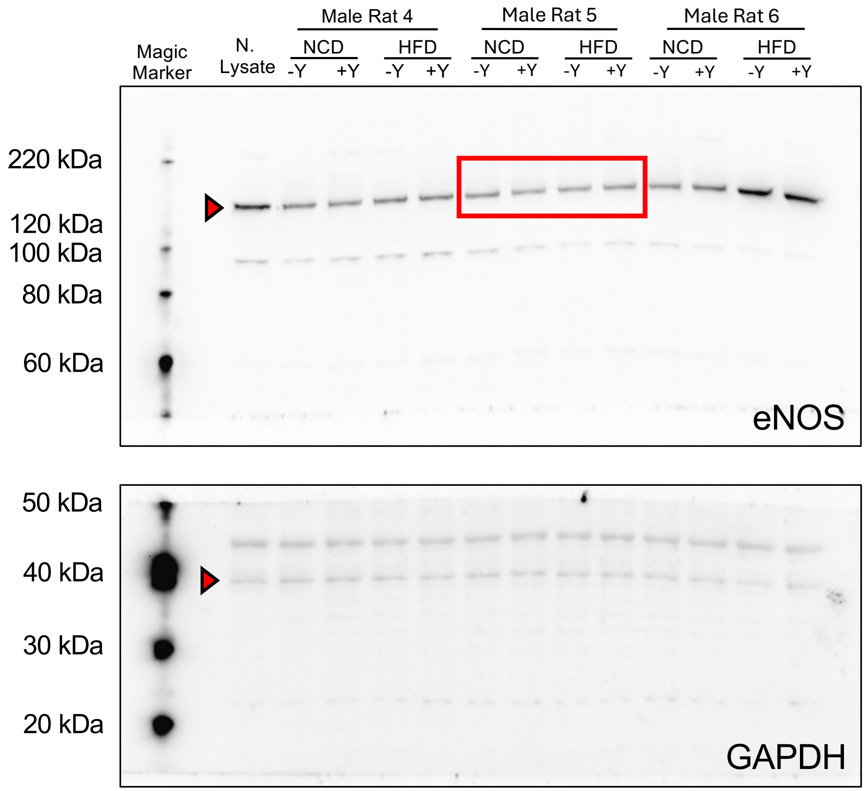
Magic Marker
N. Lysate
Male Rat 4
Male Rat 5
Male Rat 6
NCD
HFD
-Y
+Y
220 kDa
120 kDa
100 kDa
80 kDa
60 kDa
eNOS
50 kDa
40 kDa
30 kDa
20 kDa
GAPDH

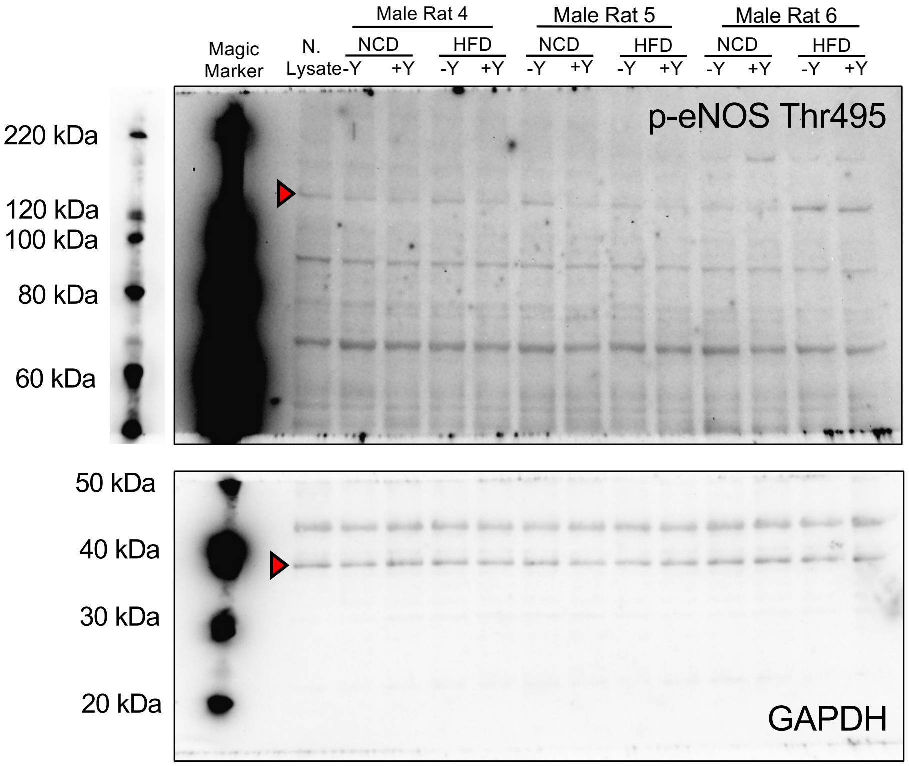
Male Rat 4
Male Rat 5
Male Rat 6
Magic Marker
N. Lysate
NCD
HFD
-Y
+Y
220 kDa
120 kDa
100 kDa
80 kDa
60 kDa
p-eNOS Thr495
50 kDa
40 kDa
30 kDa
20 kDa
GAPDH

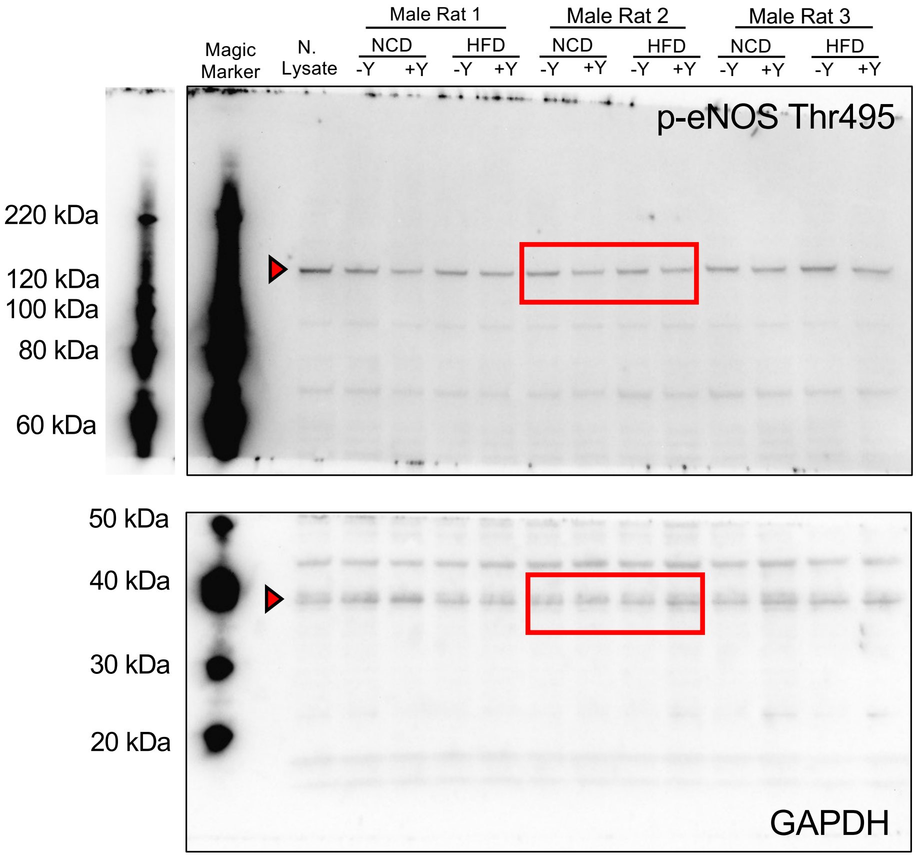
Male Rat 1
Male Rat 2
Male Rat 3
Magic Marker
N. Lysate
NCD
HFD
-Y
+Y
220 kDa
120 kDa
100 kDa
80 kDa
60 kDa
p-eNOS Thr495
50 kDa
40 kDa
30 kDa
20 kDa
GAPDH
